# Network and Evolutionary Analysis Reveals Candidate Genes of Membrane Trafficking Involved in Maize Seed Development and Immune Response

**DOI:** 10.3389/fpls.2022.883961

**Published:** 2022-06-24

**Authors:** Chunyan Zheng, Yin Yu, Guiling Deng, Hanjie Li, Faqiang Li

**Affiliations:** ^1^College of Life Sciences, South China Agricultural University, Guangzhou, China; ^2^Guangdong Provincial Key Laboratory of Protein Function and Regulation in Agricultural Organisms, College of Life Sciences, South China Agricultural University, Guangzhou, China

**Keywords:** membrane trafficking, maize, WGCNA, endosperm development, pathogen resistance, evolution, domestication, improvement

## Abstract

The plant membrane-trafficking system plays a crucial role in maintaining proper cellular functions and responding to various developmental and environmental cues. Thus far, our knowledge of the maize membrane-trafficking system is still limited. In this study, we systematically identified 479 membrane-trafficking genes from the maize genome using orthology search and studied their functions by integrating transcriptome and evolution analyses. These genes encode the components of coated vesicles, AP complexes, autophagy, ESCRTs, retromers, Rab GTPases, tethering factors, and SNAREs. The maize genes exhibited diverse but coordinated expression patterns, with 249 genes showing elevated expression in reproductive tissues. Further WGCNA analysis revealed that five COPII components and four Rab GTPases had high connectivity with protein biosynthesis during endosperm development and that eight components of autophagy, ESCRT, Rab, and SNARE were strongly co-upregulated with defense-related genes and/or with secondary metabolic processes to confer basal resistance to *Fusarium graminearum*. In addition, we identified 39 membrane-trafficking genes with strong selection signals during maize domestication and/or improvement. Among them, *ZmSec23a* and *ZmVPS37A* were selected for kernel oil production during improvement and pathogen resistance during domestication, respectively. In summary, these findings will provide important hints for future appreciation of the functions of membrane-trafficking genes in maize.

## Introduction

Like other eukaryotic organisms, plants have evolved a sophisticated membrane-trafficking system to control most, if not all, cellular events (Surpin and Raikhel, 2004). The system connects distinctive membrane-bound organelles through vesicle transport, thus maintaining proper cellular functions and responding to various developmental signals and environmental cues (Inada and Ueda, 2014; Gu et al., 2017; Wang et al., 2020b). The membrane-trafficking system is principally involved in two major routes, namely, the secretory and endocytic pathways. During the secretory pathway, proteins synthesized in the endoplasmic reticulum (ER) are transported to the Golgi apparatus, the trans-Golgi network (TGN), and ultimately reach the plasma membrane (PM) and/or apoplast or are directed to the vacuole *via* a Golgi–TGN–multivesicular endosome (MVE) pathway (Paez Valencia et al., 2016). During the endocytic pathway, PM-localized and extracellular proteins are first internalized and transported to the TGN/early endosome (EE), and then recycled back to the PM or further transported to the vacuole for degradation (Rodriguez-Furlan et al., 2019). Besides these two routes, cytoplasmic components can be degraded by a highly conserved catabolic process called autophagy, in which cargoes are engulfed by cup-shaped double-membrane vesicles termed autophagosomes and delivered to the vacuole for degradation (Marshall and Vierstra, 2018).

Both secretory and endocytic routes share conserved sequential events and involve several major molecular machineries governing these processes. First, protein cargoes are selectively recruited into the vesicles budding from the donor membrane facilitated by coat proteins (clathrin, COPI, and COPII coatomers) and adaptor protein complexes (AP-1 to AP-5). Some small GTPases, such as ADP ribosylation factor (ARF) and secretion associated and Ras-related 1 (Sar1) GTPases, are also required for this step and play critical roles in the recruitment of coat proteins (Paez Valencia et al., 2016). Different from the secretory and endocytic routes, the formation of an autophagosome requires no coat proteins and the cargoes are recognized selectively by a batch of cargo receptors (Marshall and Vierstra, 2018). Following their formation, vesicles are transported toward the target organelles along the cytoskeleton (Bhandari and Brandizzi, 2020). When reaching the target compartments, inbound vesicles are recognized and caught by tethering factors. Once tethered, vesicles are fused with target membranes mediated by soluble *N*-ethylmaleimide-sensitive-factor attachment protein receptors (SNARE) complexes. Usually, each donor and acceptor membrane contact is bridged by a specific tethering complex (Paez Valencia et al., 2016). For example, class C core vacuole/endosome tethering (CORVET) and homotypic fusion and vacuole protein sorting (HOPS) complexes mediate the tethering with the lysosome/vacuole, and conserved oligomeric Golgi (COG), a multi-subunit vesicle tethering complex, is involved in retrograde trafficking within the Golgi. Importantly, regulatory small GTPases, Rabs, regulate the steps of vesicle transport and tethering by selectively recruiting specific tethering or other effectors (Minamino and Ueda, 2019). The final step of endomembrane trafficking involves recycling the transport machinery components back to the donor membrane mediated by the retromer complex (Heucken and Ivanov, 2018). Moreover, PM proteins may also be ubiquitinated during endocytosis and sorted into intraluminal vesicles (ILVs) inside multivesicular bodies (MVBs) regulated by an endosomal sorting complex required for transport (ESCRT) machinery (Gao et al., 2017).

Through the coordination of secretory, endocytic, and autophagic routes, plants can fine-tune the levels of proteins, lipids, and other cellular components in response to various physiological and environmental changes. For example, plants can effectively modulate their protein receptors, transporters, and channels at the PM to facilitate environmental sensing, nutrient uptake, and intercellular communication. In the past decades, studies with *Arabidopsis* and rice have revealed the important roles of endomembrane trafficking in coordinating plant development and in response to various biotic and abiotic environmental stresses (Inada and Ueda, 2014; Gu et al., 2017; Wang et al., 2020b). The trafficking and deposition of seed storage proteins (SSPs) offer an outstanding example for us to appreciate how an endomembrane system changes morphologically and functionally to accommodate seed development (Zheng et al., 2022). The *Arabidopsis* major SSPs (2S albumins and 12S globulins) are first synthesized in the ER and then transported to the protein storage vacuoles (PSVs) *via* the ER–Golgi–TGN–MVE pathway in the embryo. In contrast, rice SSPs are predominantly accumulated in the sub-aleurone layer of endosperm (He et al., 2021). Once synthesized, rice SSPs can either remain inside the ER as protein bodies (PBs) or be exported to specialized PSVs. During seed development in both *Arabidopsis* and rice, numerous components of coat complexes, Rab GTPases, tethering factors, SNAREs, and retromers appear to participate in SSP transport because SSPs are often missorted to the apoplast when these factors are absent (He et al., 2021; Zheng et al., 2022).

The endomembrane-trafficking system also plays a vital role in the rapid response to pathogen infection. Upon infection, PM-localized immune receptors undergo endocytosis while antimicrobial peptides and cell wall components are secreted to the pathogen infection sites (Inada and Ueda, 2014; Gu et al., 2017). For example, the SYP1 group of Qa-SNARE localizes to the PM and is responsible for the membrane fusion with the PM. Studies with *Arabidopsis pen1* and Barley (*Hordeum vulgare*) *ror2-1* mutants have revealed the contribution of SYP121 to the penetration resistance against non-adapted powdery mildew fungi (Collins et al., 2003; Kwon et al., 2008). Tobacco (*Nicotiana benthamiana*) SYP132 was reported to be involved in multiple forms of plant defenses and the secretion of pathogenesis-related protein PR1a (Kalde et al., 2007). Moreover, the secretion of PR1 in *Arabidopsis* was also found to be negatively regulated by SNAREs BET12 and MEMB12, which are involved in protein trafficking in the early secretory pathway (Chung et al., 2018). As a unique catabolic process, autophagy plays multiple roles during plant–microbe interactions, including pathogen clearance, execution of pathogen-induced cell death, and modulation of innate immune signaling pathways (Hofius et al., 2017). Therefore, plant autophagy machinery is also a pivotal target of pathogens during infection.

Until now, our knowledge about the plant endomembrane-trafficking system has mainly been obtained from *Arabidopsis*, and the studies with rice allowed us to appreciate the contributions of this system to SSP transport during seed development (He et al., 2021). Although maize is the leading grain crop worldwide, few studies on its membrane-trafficking system have been reported. For example, overexpression of *ZmVPS29*, a retromer complex component, conferred a slender kernel morphology (Chen et al., 2020b), and disruption of supernumerary aleurone 1 (SAL1, an ESCRT component) and Discolored1 (DSC1, an ADP-ribosylation factor GTPase) resulted in abnormal seed development (Shen et al., 2003; Takacs et al., 2012). The importance of the membrane-trafficking system was also revealed by a comparative genomic analysis of wild, landrace, and modern maize (Hufford et al., 2012), in which 31 factors involved in membrane trafficking were found to be selected during maize domestication and/or improvement, implying their strong connections with important agronomical traits. Therefore, it is important to understand the biological functions of maize membrane-trafficking factors. Here, the maize membrane-trafficking genes were identified using bioinformatic methods and publicly available data and characterized by the integration of co-expression network analysis and evolution analysis. These findings will provide important hints for future research to unravel the roles of these genes in maize development and responding to biotic and abiotic stresses.

## Materials and Methods

### Identification of Membrane-Trafficking Genes

Two methods were used to identify maize membrane-trafficking genes. First, the maize genome and protein databases were downloaded from the Ensembl Plants database^1^ (B73 RefGen version 3 and version 4). The protein sequences of *Arabidopsis* membrane-trafficking components were retrieved from the TAIR10 database^2^ and employed as queries to search against local databases using BLASTP and TBLASTN methods (E value ≤ 1.0e-30). The orthology relationships between maize and *Arabidopsis* genes were further confirmed using the orthologs identification tool in the MCENet server^3^ (Tian et al., 2018). Second, the hidden Markov model (HMM) profiles of available membrane-trafficking components were retrieved from Pfam^4^ and used to search against the local maize protein database using the BLASTP method. The redundant sequences obtained from both methods were manually removed.

### Phylogenetic Analysis and Conserved Domains of the Vesicle-Trafficking Proteins

Full-length protein sequences of maize and *Arabidopsis* membrane-trafficking proteins were aligned using ClustalW with default parameters, and phylogenetic trees were generated by MEGA (version 6.0) software using the neighbor-joining method with 1,000 bootstrap replications. The resulting Newick trees were visualized with the Evolview-v2 online server.^5^ All the conserved domains of membrane-trafficking proteins were identified using the NCBI Batch CD-Search database^6^ (Lu et al., 2020) and Pfam website with default parameters. The conserved domains were visualized with TBtools (Chen et al., 2020a).

### Construction of Gene Co-expression Network Analysis

An R package, WGCNA, was used to construct gene co-expression networks (Langfelder and Horvath, 2008). For network analysis, two transcriptomic datasets, B73 different tissue RNA-seq data and Illumina HiSeq data (GEO accession: GSE92448), were collected and filtered to remove lower-quality reads and reads with expression variance of <20% (Stelpflug et al., 2016; Kebede et al., 2018). In the weighted gene network analysis, each node represented a gene, and the edge was determined by the similarity of paired genes. Pearson correlation coefficients were calculated based on the similarity of paired gene expression and then transformed into an adjacency matrix of connection strength, which was used to calculate the topographical overlap matrix (TOM). A 1-TOM was calculated using hierarchical clustering, which defined gene modules corresponding to the basis of dissimilarity of gene connectivity. The total connectivity and intramodular connectivity, KME (for eigengene-based connectivity), and the KME P-value were calculated. The eigengene value for each module (ME) with phenotypic traits was calculated, and the most significant module was considered the core module. Each module had a unique color. Gene connectivity in modules balanced the number of edges of a node and was further visualized in Gephi-0.9.2 software.^7^ Gene Ontology (GO) categories of co-expression interactions with membrane-trafficking genes in the module were performed using R 3.6.4 packages clusterProfiler (Yu et al., 2012).

### Gene Expression Pattern Analysis

To analyze the spatiotemporal expression pattern of maize membrane-trafficking genes, the expression data were retrieved from the previously described RNA-seq dataset (Stelpflug et al., 2016). To analyze the expression level of *ZmSec23a* in the seeds of regular and high oil inbred lines, the expression data in 15 DAP kernels of 368 maize inbred lines were downloaded from Yan’s lab website^8^ (Fu et al., 2013). The expression heatmaps were generated using the R.3.6.4 platform.^9^

### Identification of Membrane-Trafficking Genes Selected During Maize Domestication and Improvement

The single nucleotide polymorphism (SNP) data were downloaded from the HapMapV3 study for 1,210 maize lines (HapMapV3.2.1)^10^ (Bukowski et al., 2018). After filtering out SNPs with ≤50% missing data, 28,803,913 SNPs were obtained. Moreover, 3.6 million SNPs were retrieved from the 368 RNA-seq association mapping panel, which include 32 high oil lines^11^ (Fu et al., 2013). After filtering with a minor allele frequency of ≥5% using the TASSEL software (Bradbury et al., 2007), 557,977 higher-quality SNPs were obtained.

The identification of genomic selective sweep regions and selected vesicle membrane-trafficking genes during maize domestication and improvement was conducted as described in the previous report (Lin et al., 2014). Briefly, the nucleotide diversity (π) ratio and the fixation index (*Fst*) during domestication (teosinte vs. landrace) and improvement (landrace vs. improved) processes were calculated using VCFtools (Danecek et al., 2011), with a window size of 10 kb and step size of 2 kb. The top 5% of π ratio (1.55 and 1.22 for domestication and improvement, respectively) and the top 5% *Fst* values (0.25 and 0.089 for domestication and improvement, respectively) were set as the thresholds for putative selection signals. When the values of π or *Fst* of the genomic region exceeded the threshold, the detected membrane-trafficking genes in the region were defined as “domestication” or “improvement.”

### Linkage Disequilibrium Analysis and Haplotype Analysis Within Genes

Single nucleotide polymorphisms (MAFs ≥ 0.05) and full-length DNA sequences of candidate genes were extracted from wild teosintes, landraces, improved cultivars, and high oil accessions. These SNPs were used to calculate the square values of the correlation coefficient (*r*^2^) between each SNP pair to measure the linkage disequilibrium (LD) using HaploView version 4.2 (Barrett et al., 2005). Haplotypes were generated using DnaSP version 5 (Librado and Rozas, 2009). For haplotype network analysis of *ZmSec23a* (*Zm00001d009150*), a sample of 20 teosintes, 22 landraces, 58 maize inbred lines, 23 high oil materials, and 345 regular inbred lines was used to construct the haplotype network using POPART version 1.7 (Leigh and Bryant, 2015).

### *Cis*-Element Analysis

Putative promoter regions containing 6,000 bp of 5′ genomic sequences immediately upstream of the start code of the *ZmVPS37A* gene were extracted from the teosinte and maize genome database^12^ and were submitted to PlantCARE^13^ for *cis*-element analysis.

### Plant Growth and Fungal Strains Infection

Teosinte (*Zea mays* ssp. *Parviglumis*, accessions: W71-2), landrace (303WX), and B73 inbred lines were grown in a greenhouse at 25°C under a light/dark 16/8-h photoperiod. The *Fusarium graminearum* wild-type strain PH-1 used in this study was cultured at 25°C on a carnation leaf agar for 1 week and then transferred and incubated in liquid carboxymethyl cellulose (CMC) medium with gentle shaking (100 rpm) for three more days. The conidium was subsequently collected by filtering through the two layers of Miracloth (Millipore, 475855) and the number of conidia was counted using a hemocytometer. For the inoculation assay, in the stalk of maize V2 stage seedlings, a hole was created 2 cm above the soil using a sterile toothpick, 40 μl suspension conidia (1 × 10^6^ conidia/ml) or ddH_2_O (mock) was injected into the hole with a needleless syringe, and then it was placed in a greenhouse for 7 days (70% humidity, 25°C, a light/dark 16/8-h photoperiod).

### Quantitative Real-Time Polymerase Chain Reaction

Maize stalk tissues were collected and frozen immediately in liquid nitrogen, and total RNA samples were isolated using the Plant RNA extraction kit (TIANDZ, Inc., Beijing, China). One microgram of total RNA was converted to cDNA using HiScript II RT SuperMix for qPCR (Vazyme, Nanjing, China) according to the manufacturer’s instructions. qRT-PCR reactions were performed with the Bio-Rad CFX96™ real-time PCR system using ChamQ Universal SYBR qPCR Master Mix (Vazyme, Nanjing, China). Maize *Ubc9* was used as an internal control (forward primer, 5′-acgaaggtcttccatccaaacatc-3′; reverse primer, 5′-gtttcatgggcaacaccacaatcg-3′), and the relative expression levels for target genes were calculated using the 2^–ΔΔCt^ method. qRT-PCRs were all performed with three biological replicates. The primers used for *ZmVPS37A* are as follows: *ZmVPS37A*-F 5′-gagctggagatttcctctcttc-3′ and *ZmVPS37A*-R 5′-gaagactgcggactatctgaagc-3′.

## Results

### Genome-Wide Identification of Membrane-Trafficking Factors in Maize

**FIGURE 1:**
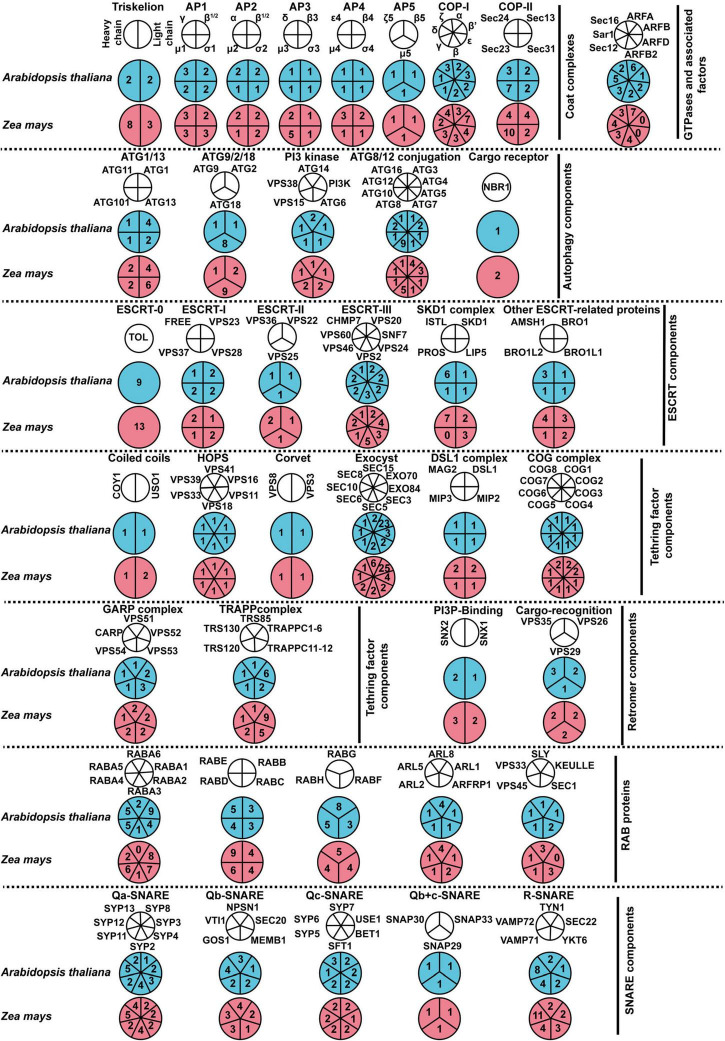
An overview of endomembrane-trafficking components in *Arabidopsis thaliana* and *Zea mays*. Coulson plot depicting components of coat complexes, autophagy machinery, ESCRT machinery, tethering factors, retromer proteins, Rab proteins, and SNARE proteins in *A. thaliana* and *Z. mays*. Legends at the top of each column refer to the subunits of complexes. Numbers indicate relevant paralog counts. Homologs of these factors were identified and listed in SupplementaryDatasets 1–7.

Prior studies by Paul et al. (2014) and Li et al. (2015) defined the maize genomic loci that encode components of secretory and endocytic pathways and autophagy pathways, respectively. Here, with the improved maize genome sequence available [RefGen_v4, Jiao et al. (2012)], we exhaustively search for membrane-trafficking components using BLASTP and TBLASTN^14^ with the full-length amino acid sequences of *Arabidopsis* membrane-trafficking factors as baits (Bassham et al., 2008; Paul et al., 2014; Gao et al., 2017). By sequence alignment and phylogenetic and domain analyses, the membrane-trafficking genes in the maize genome are defined (Figure 1, Supplementary Figures 1–9, and Supplementary Datasets 1–7). Totally, we obtained 479 maize genes involved in membrane trafficking, including 11 genes encoding components for clathrin-coated vesicle (CCV), 35 for AP1-5 complexes, 26 for COP-I complex, 30 for COP-II complex, 10 for ARF GTPases, 52 for autophagy machinery, 63 for ESCRTs machinery, 96 for tethering complexes, 11 for retromer complex, 76 factors for Rab GTPase protein family, and 69 for SNARE protein family.

The factors are highly comparable in their protein length and the number of orthologs between maize and *Arabidopsis* (Supplementary Figures 1–9 and Supplementary Datasets 1–7). However, we observed a significantly higher copy number for certain factors in maize than in *Arabidopsis*. For example, there are seven, four, and three gene copies for the B-COP β’ and ε subunits, and F-COP γ subunit, respectively, while there are only three, two, and one genes for the relevant *Arabidopsis* factors. Another clear example is the genes encoding clathrin heavy chain proteins, with eight and two gene copies in maize and *Arabidopsis* genomes, respectively. Notably, an interesting exception is that *Arabidopsis* possesses a slightly expanded set of Rab11 (RabA), with 26 paralogs as compared to 24 paralogs in the maize genome (Supplementary Figure 8 and Supplementary Dataset 6). Moreover, we could not detect any orthologs of PROS (an ESCRT component) and RABA6 protein in maize (Supplementary Datasets 3, 6).

Domain architecture is critical for protein function and is therefore mostly retained by orthologs during evolution for their function performance. Thus, we compared the domain architectures of maize factors with their relevant *Arabidopsis* components as an additional criterion to assign orthology. Using NCBI Conserved Domain (CDD^15^) and Pfam website,^16^ we observed that 81.84% (392/479) of maize factors possess the same domain architecture as their *Arabidopsis* orthologs, which indicates that the membrane-trafficking system is largely conserved in monocotyledonous and dicotyledonous plants. In addition, we found that 6.47% (31/479) of maize orthologous genes are predicted to encode truncated proteins with defective domain structures. For examples, four genes [*Zm00001d009915* (*ZmCHC3a*), *Zm00001d026086* (*ZmCHC3b*), *Zm00001d021067* (*ZmCHC4a*), and *Zm00001d012897* (*ZmCHC5b*)] are predicted to encode truncated clathrin heavy chain protein (Supplementary Figure 1), and two genes [*Zm00001d013458* (*ZmATG3b*) and *Zm00001d003156* (*ZmATG3d*)] are predicted to encode truncated autophagy component ATG3 (Supplementary Figure 4). Further gene expression analysis with MaizeGDB^17^ revealed that no cDNAs have been detected for these six genes, indicating that they are likely pseudogenes (data not shown).

In some cases, we observed the presence of additional domains in some maize factors when compared with their *Arabidopsis* orthologs, e.g., for SNARE protein SYP3 or ESCRT component SNF7. *Arabidopsis* Qa-SNARE protein AtSYP31 (AT5G05760) and AtSYP32 (AT3G24350) contain a t-SNARE domain at their C-terminal regions, while an additional syntaxin-5 domain is detected at the N-terminal regions of maize orthologs ZmSYP31 (Zm00001d008758) and ZmSYP32 (Zm00001d039614) (Supplementary Figure 9). A similar scenario is also detected for one of the maize ESCRT SNF7 proteins (Zm00001d044480), which contains an additional leucine-rich repeat N-terminal domain besides the C-terminal SNF7 domain (Supplementary Figure 5). These extra domains might provide another layer of regulatory feature for these maize membrane-trafficking factors.

### RNA-Seq Analysis of Maize Membrane-Trafficking Gene Expression

**FIGURE 2:**
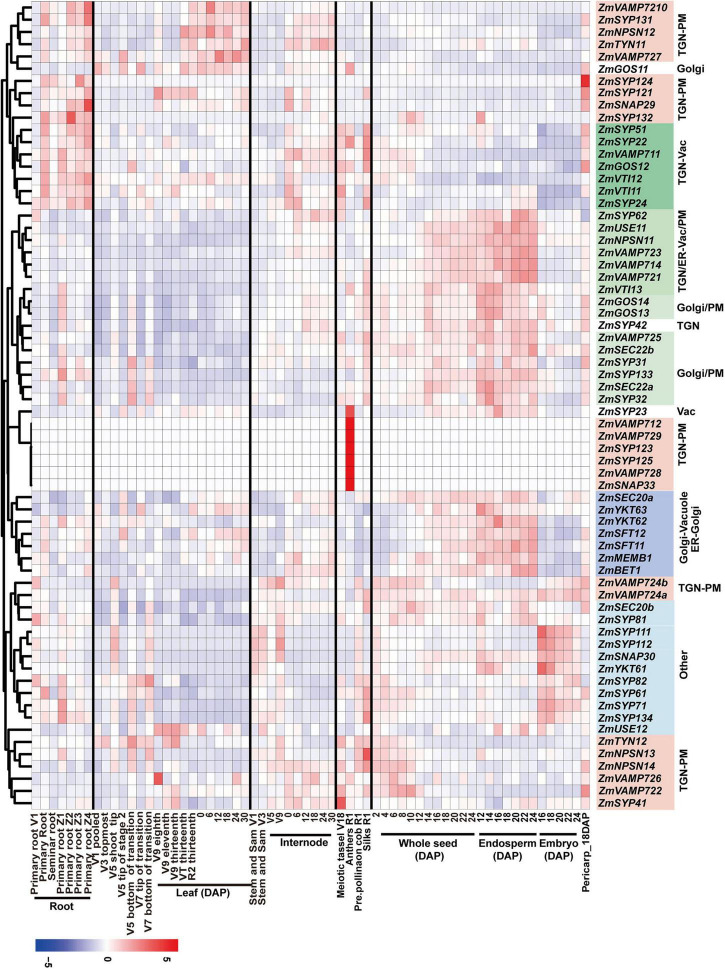
The spatiotemporal expression profiles of maize genes encoding SNARE proteins. The expression patterns of SNARE-encoding genes are analyzed based on the reads per kilobase per one million reads (RPKM) of 64 maize tissue-specific samples. The blue, white, and red colors indicate the low, medium, and high gene expression values, respectively.

To investigate the spatiotemporal expression patterns of maize membrane-trafficking genes, we performed a gene co-expression (clustering) analysis using a previously described RNA-seq dataset derived from 64 different tissues/stages of the maize inbred line B73 (Stelpflug et al., 2016; Figure 2 and Supplementary Figures 10–16). We observed 3–12 clusters in all protein complexes or families analyzed, indicating that the expression profiles of these genes varied considerably. Notably, with few exceptions (e.g., *ZmGOS11*), most genes encoding SNARE proteins are grouped into nine clusters, which largely coincide with their subcellular localization (Figure 2). The first and last clusters mostly contained genes encoding TGN/PM-localized SNAREs, including *ZmSYP1*, *ZmVAMP72*, *ZmNPSN1*, and *ZmTYN11*, but showed distinct expression patterns, indicating that these two sets of *SNARE* genes might have tissue-specific functions. The genes in the second cluster (TGN-Vac) displayed elevated expression in root and some reproductive tissues (silk and tassel), whereas the genes in the third (TGN-vac or PM), fourth (Golgi), and sixth (ER-Golgi-Vac) clusters showed enhanced expression in endosperm but not in embryo. Interestingly, we also detected increased mRNA abundance in endosperm for multiple membrane-trafficking genes (175 loci) other than SNAREs (Supplementary Figures 10–16). It was similar to the earlier observation for maize autophagy machinery (Li et al., 2015), indicating that the membrane-trafficking system, including the autophagy pathway, might participate in the maturation of maize endosperm during seed development. The fifth cluster contained seven *SNARE* genes (*ZmSNAP33*, *ZmVAMP728*, *ZmVAMP729*, *ZmVAMP712*, *ZmSYP125*, *ZmSYP123*, and *ZmSYP23*), which all expressed preferentially in anthers above all other tissues, implying their roles during anther and/or pollen development.

Besides the analysis of spatiotemporal expression patterns, our RNA-seq analyses also detected differential expression at the isoform level for multiple membrane-trafficking genes. For example, VAMP72, which is a subgroup of VAMP7 specific to green plants and is largely involved in trafficking at the PM, had eleven isoforms in the maize genome and could be subdivided into five groups based on their expression patterns (Figure 2). The first group, encompassing *ZmVAMP727* and *ZmVAMP7210*, displayed elevated expression in root and leaf tissues. However, the second group, containing *ZmVAMP721*, *ZmVAMP723*, and *ZmVAMP725*, had enhanced expression patterns in whole seed and endosperm tissues, suggesting a role in regulating seed development. The third group included *ZmVAMP728* and *ZmVAMP729*; they were expressed exclusively in anther tissue, implying their specific role in pollen development. The fourth group contained *ZmVAMP722* and *ZmVAMP726*, which were expressed preferentially at the very early stage of seed development. The last group (*ZmVAMP724a* and *ZmVAMP724b*) had ubiquitous expression patterns.

### Identification of Membrane-Trafficking Genes Involving Seed Development

Our spatiotemporal expression analysis revealed that 257 membrane-trafficking genes displayed elevated expression in the endosperm, embryo, or whole seed tissues, suggesting that they might play important roles in seed development (Figure 2 and Supplementary Figures 10–16). To further investigate the contributions of these genes in maize seed development, we performed a weighted gene co-expression network analysis (WGCNA) to analyze the co-expression relationships among 16,888 differentially expressed genes identified from the above-mentioned RNA-seq dataset (Stelpflug et al., 2016). In total, 27 color-coded gene modules were identified according to the similarity of gene expression patterns (Supplementary Figure 17A and Supplementary Table 1). The module-tissue correlation analysis showed that modules turquoise and yellow positively correlated with early and late endosperm development, respectively (*r* = 0.36, *p* = 9.45e–4; *r* = 0.39, *p* = 3.44e–4, respectively) (Supplementary Figure 17B). The genes of module turquoise peaked at the endosperm of 12 DAP (days after pollination), whereas those in module yellow were expressed highly at the endosperm of 24 DAP (Supplementary Figures 17C,D). Moreover, module black was correlated with embryo development (*r* = 0.66, *p* = 2.46e–11), and module dark red was correlated with early seed development (*r* = 0.56, *p* = 5.56e–8).

**FIGURE 3:**
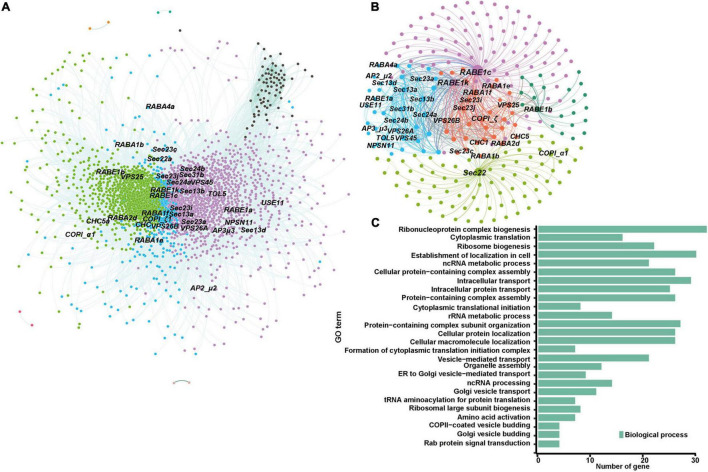
Co-expression of membrane-trafficking genes during maize seed development. Nodes are color coded based on the consensus modules identified using the R language WGCNA package. Edges are constructed between genes with a correlation coefficient (*r*) > 0.2. **(A)** Co-expression network visualization for module turquoise identified in Supplementary Figure 17. **(B)** The subnetwork with membrane-trafficking genes in module turquoise for visualization (*r* > 0.2). **(C)** Gene ontology (GO) enrichment analysis of the subnetwork co-expressed genes.

In early endosperm-specific module turquoise, 114 genes encoding membrane-trafficking factors were identified among 3,850 annotated genes, including 18 for CCV and AP complexes, 15 for COPI complex, 15 for COPII complex, three ARF GTPases, six for ESCRT complex, three for retromer complex, 30 for tethering factors, 16 for Rab GTPases, and 8 for SNARE proteins (Supplementary Table 1). Further analysis of the subnetwork harboring components with a weighted value over 0.2 revealed that genes encoding five COPII components (ZmSec13a, ZmSec13b, ZmSec24a, ZmSec23i, and ZmSec23j), four Rab GTPases (ZmRABA1f, ZmRABE1b, ZmRABE1c, and ZmRABE1k), one Golgi/PM localized SNARE protein (ZmSec22), and one ESCRT-II component (ZmVPS25) have higher connectivity with other genes (Figures 3A,B). The COPII complex is responsible for the transport of newly synthesized protein from ER to *cis*-Golgi, and RabE GTPases are well-known regulators for vesicle transport from the Golgi apparatus to the PM (Speth et al., 2009). The GO enrichment analysis revealed that the genes involved in protein biosynthesis and assembly, ribosome biogenesis, and translation apparatus are overrepresented (Figure 3C), suggesting the role(s) of these membrane-trafficking components in the delivery of nascent biosynthesized proteins during early endosperm development.

### Identification of Membrane-Trafficking Components Responsible for Pathogen Defense in Maize

To investigate the membrane-trafficking genes responsible for pathogen defense during Gibberella ear rot disease caused by *F. graminearum*, we also performed a WGCNA to identify gene modules between resistance inbred line CO441 and susceptible line B73 (Kebede et al., 2018). In total, 15 modules were identified among 12,528 differential expression genes across eight experimental conditions (Supplementary Figure 18A and Supplementary Table 2). According to the module-trait correlation analysis (Supplementary Figure 18B), modules red and magenta showed a highly positive correlation with disease defense of resistance line CO441 (*r* = 0.54, *p* = 0.04; *r* = 0.57, *p* = 0.03, respectively). The genes within module red were highly expressed in resistance line CO441 at 1–2 days after fungi infection (Supplementary Figure 18C). In contrast, genes within module green were highly expressed in susceptible line B73 (*r* = 0.7, *p* = 0.04) and peaked at 2 days after fungi infection (Supplementary Figure 18D).

In module green, four membrane-trafficking genes, which encode two ESCRT components (ZmTOL9 and ZmISTL6), one SNARE protein ZmSYP121, and a SNARE-interacting protein ZmSec1b were identified among 603 annotated genes with a weighted value over 0.3 (Supplementary Figures 19A,B). These genes were strongly co-upregulated with several defense genes in B73 2 days after fungi infection (Supplementary Figure 19C). Within the subnetwork containing these four genes, genes involved in defense, SA biosynthesis, jasmonic acid signaling, and amino acid biological process are overrepresented (Supplementary Figure 19D), implying the role of these four factors in response to pathogen infection. The identification of *ZmSyp121* here further confirmed its conserved role in pathogen defense in both monocotyledonous and dicotyledonous plants (Collins et al., 2003).

**FIGURE 4:**
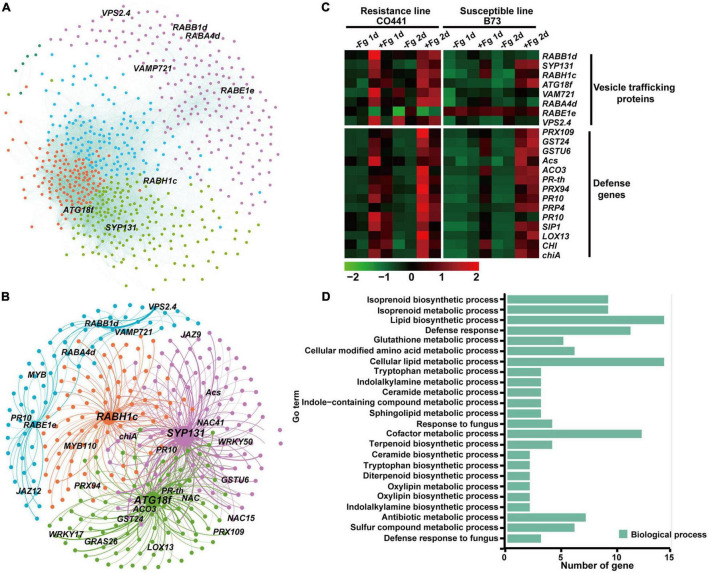
Co-expression of membrane-trafficking genes responding to Gibberella ear rot disease in two maize inbred lines. **(A)** Co-expression network of module red. Nodes are color coded based on the consensus modules identified using the R language WGCNA package. Edges are constructed between genes with a correlation coefficient (*r*) > 0.15. **(B)** The subnetwork with membrane-trafficking genes for visualization in module red. **(C)** Expression profiles of defense and membrane-trafficking genes in module red upon Gibberella infection. The values in red and green indicate gene increases and decreases in expression, respectively. **(D)** Gene ontology (GO) enrichment analysis of co-expressed genes of module red.

We then analyzed module red and identified eight membrane-trafficking genes among 671 annotated genes (weighted value > 0.15) (Figures 4A,B). These factors all showed a stronger and more rapid gene expression in resistant line CO441 than in B73 upon pathogen infection, along with many defense genes, such as *PR1* and *PR10* (Figure 4C). Among these genes, those encoding SNARE protein SYP131, Rab GTPase RABH1c, and autophagy component ATG18f showed a higher degree of connectivity (>10). The *Arabidopsis* orthologs of SYP131 and RabH1b have been reported to be involved in the vesicle trafficking of callose (Gamir et al., 2018; He et al., 2018). The identification of *ZmSYP131* and *ZmRABH1c* here suggested that maize orthologs might have a similar function in callose deposition upon fungi infection. The identification of *ZmATG18f* also suggested the role of autophagy in the pathogen defense of maize. Further, GO analysis of the subnetwork containing these eight genes revealed an enrichment of defense genes (Figure 4D). Moreover, GO terms related to the biological process of biotic stress such as lipid biosynthetic process, isoprenoid biosynthetic process, and trypotophon metabolic process were also specifically enriched, indicating that membrane-trafficking factors likely coordinated with the defense-related metabolic processes and transported defense proteins and antimicrobial metabolites to the infected regions. In summary, the WGCNA analysis above revealed that the maize genes encoding membrane-trafficking factors were upregulated together with defense-related genes, which conferred basal resistance to *F. graminearum* in B73, but also with genes involved in various secondary metabolic processes to further improve resistance capability in CO441 line.

### Multiple Membrane-Trafficking Genes Are Selected During Maize Domestication and Improvement

**FIGURE 5:**
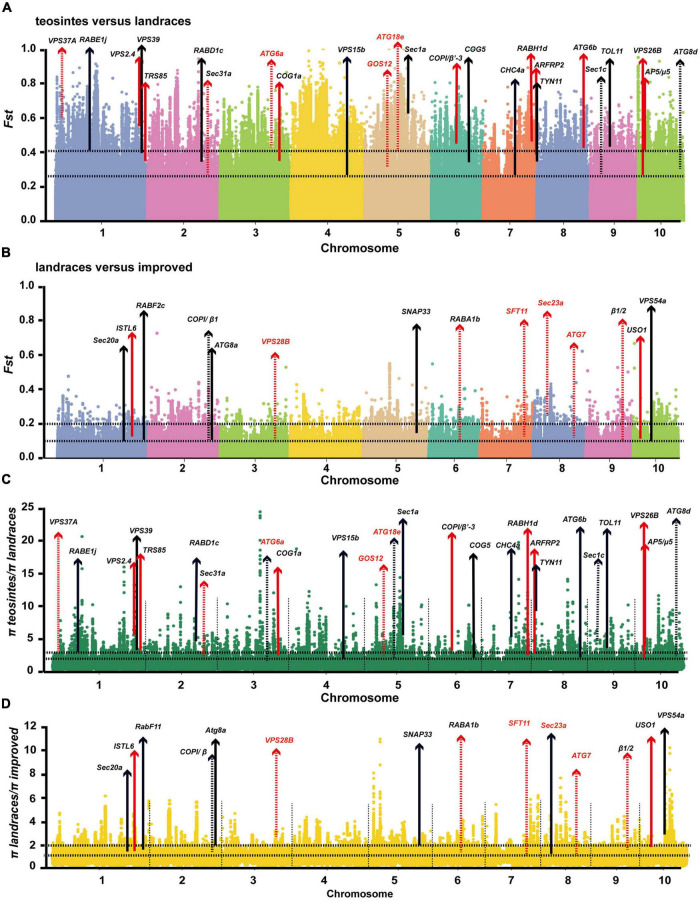
Profiling of selected membrane-trafficking genes during maize domestication and improvement. **(A–D)** Genome-wide selective signals (*Fst* and π ratio) analysis of teosinte, landrace, and improved using a 20-kb sliding window and 10-kb steps. The chromosome numbers are set along the x-axis. The top and bottom horizontal black dashed lines in each panel represent the top 1% (*Fst* ≥ 0.41; π teosinte/π landrace ≥ 2.56; *Fst* ≥ 0.14; π landrace/π improved ≥ 1.99) and top 5% (*Fst* ≥ 0.26; π teosinte/π landrace ≥ 1.55; *Fst* ≥ 0.08; π landrace/π improved ≥ 1.39) of cutoffs, respectively. Arrows indicate that membrane trafficking genes were selected during maize domestication and improvement. Red arrows represent these genes that have also been identified in previous evolutionary analyses (Hufford et al., 2012). Dashed lines indicate domestication or improvement of genes falling within the selected region. Solid lines indicate domestication and improvement candidates.

The identification of selected genetic loci during domestication and improvement of crops is important for breeding elite varieties and can further facilitate appreciation of the contributions of selected loci to important agronomic traits (Zhou et al., 2015). Molecular evidence for selective sweeps on multiple membrane-trafficking genes during maize domestication and improvement has been reported in several previous studies (Hufford et al., 2012; Jiao et al., 2012; Liu et al., 2015). With more comprehensive and better annotated B73 genome datasets and the complete list of membrane-trafficking genes, we searched the larger and more diverse maize HapMap 3 genotype dataset (Bukowski et al., 2018), which covers 20 wild relatives (*Zea mays* spp. *Parviglumis*), 23 landraces, and 59 improved cultivars (Supplementary Table 3), for genomic regions showing positive selection signals during domestication and improvement through statistical analyses of high population differentiation (*Fst*) and low level of nucleotide diversity (π) (Table 1). The top 5% of *Fst* and π ratio were set as thresholds to identify potentially selected regions. During maize domestication (teosinte vs. landrace), 15,760 windows were found as candidate regions (*Fst* ≥ 0.26; π teosinte/π landrace ≥ 1.55). A total of 25 out of 479 membrane-trafficking genes were found to be associated with domestication (Figures 5A,C and Table 1), including five genes encoding components of the autophagy machinery, three for ESCRT complex, four for Coat and AP complexes, one for retromer complex, four for tethering factors, six for ARF and Rab GTPases, and two for SNARE proteins. In the comparison of maize landrace and improved populations, 14,729 slide windows were revealed by two methods (*Fst* ≥ 0.08; π landrace/π improved ≥ 1.39). Similarly, a total of 14 membrane-trafficking genes were identified to be improvement genes (Figures 5B,D and Table 1), including two for autophagy factors, three for SNARE proteins, two for Rab GTPases, two for tethering factors, three for coat complexes, and two for ESCRT components.

**TABLE 1:** Membrane-trafficking genes selected during maize domestication and improvement.

	*Zea mays* ID	Chr.	Position	Top 5% *Fst*	Top 5% π ratio	Annotation
Domestication	*Zm00001d052028^a^*	4	173834001-173850000	0.32	1.771	*ZmVPS15b*
	*Zm00001d022584^a^*	7	174832000-174858000	0.361	10.577	*ZmTYN11*
	*Zm00001d023738^a^*	10	17782000-178400000	0.479	3.832	ZmVPS26B*
	*Zm00001d021067^a^*	7	135926000-136002000	0.489	4.348	*ZmCHC4a*
	*Zm00001d046020^a^*	9	54474000-54620000	0.39	3.139	*ZmTOL11*
	*Zm00001d005215^a^*	2	159788000-159848000	0.409	2.889	*ZmRABD1c*
	*Zm00001d015890^a^*	5	126234000-126294000	0.645	2.412	*ZmSec1a*
	*GRMZM2G360867^a^*	1	72404000-72516000	0.441	3.79	*ZmRABE1j*
	*Zm00001d011984^a^*	8	160366000-160470000	0.425	2.931	ZmATG6b*
	*Zm00001d022172^a^*	7	166126000-166198000	0.567	4.651	ZmARFRP2*
	*Zm00001d027962^a^*	1	18046000-18130000	0.589	2.154	ZmVPS37A*
	*Zm00001d032710^a^*	1	232184000-232276000	0.614	7.41	ZmVPS2.4*
	*Zm00001d021551^a^*	7	150440000-150572000	0.444	2.639	ZmRabH1d*
	*Zm00001d023910^a^*	10	26982000-27084000	0.525	2.639	*ZmAP5/*μ5*
	*Zm00001d036161^a^*	6	72344000-72418000	0.515	2.640	*Zm*β’3-COPI*
	*Zm00001d043288^a^*	3	191668000-191678000	0.341	1.895	ZmCOG1a*
	*Zm00001d034364^a^*	1	285830000-285840000	0.309	2.784	ZmTRS85*
	*Zm00001d033776^a^*	1	268656000-268668000	0.400	2.852	*ZmVPS39*
	*Zm00001d038059^a^*	6	142706000-142716000	0.352	1.551	*ZmCOG5*
	*Zm00001d042303^b^*	3	158480001-158500000	0.487	1.535	ZmATG6a*
	*Zm00001d015102^b^*	5	73180001-73284000	0.335	1.825	ZmGOS12*
	*Zm00001d007075^b^*	2	215490001-215528000	0.269	1.684	ZmSec31a*
	*Zm00001d018355^b^*	5	213270001-213318000	0.442	2.062	ZmATG18e*
	*Zm00001d024239^b^*	10	59888000-59978000	0.493	2.170	*ZmATG8d*
	*Zm00001d045434^b^*	9	22254001-22270000	0.299	6.133	*ZmSec1c*
Improvement	*Zm00001d033058^a^*	1	245198000-245304000	0.155	1.874	*ZmSec20a*
	*Zm00001d016686^a^*	5	168500000-168562000	0.141	2.127	*ZmSNAP33*
	*Zm00001d034949^a^*	1	300880000-301008000	0.16	2.16	*ZmRABF2c*
	*Zm00001d006474^a^*	2	203384000-203406000	0.157	2.154	*ZmATG8a*
	*Zm00001d009150^a^*	8	38490000-38546000	0.211	1.721	ZmSec23a*
	*Zm00001d033714^a^*	1	267240000-267272000	0.126	1.565	ZmISTL6*
	*Zm00001d025044^a^*	10	100866000-100876000	0.117	3.101	*ZmVPS54a*
	*Zm00001d023867^a^*	10	24958000-24968000	0.156	1.985	ZmUSO1*
	*Zm00001d043269^b^*	3	191006000-191018000	0.092	2.272	ZmVPS28B*
	*Zm00001d047464^b^*	9	128998000-129008000	0.117	1.496	ZmAP1/2B2’*
	*Zm00001d011347^b^*	8	142642001-142702000	0.155	1.413	ZmATG7*
	*Zm00001d021913^b^*	7	160350001-160370000	0.137	1.143	ZmSFT11*
	*Zm00001d037004^b^*	6	105584001-105610000	0.129	1.359	ZmRABA1b*
	*Zm00001d004807^b^*	2	136600000-136772000	0.147	1.537	*Zm*β*1-COP-I*

*Chr, chromosome number. ^a,b^Denote the gene status of identified loci either as a candidate gene or in selected regions, respectively. Asterisks indicate the loci that have been identified in previous analyses. GRMZM2G360867 is not available in Z. mays RefGen_V4.*

Out of the 39 selected membrane-trafficking genes identified in our study, 22 have been identified in previous evolutionary analyses (Hufford et al., 2012; Jiao et al., 2012; Liu et al., 2015). Moreover, nine out of 39 selected membrane-trafficking genes were detected by QTL mapping or genome-wide association studies (Supplementary Table 4), which further implicates the potential roles of these genes in maize development and biotic and/or abiotic stress responses. For example, four of them are implicated in biotic stress responses [*Zm00001d042303* (*ZmATG6a*), *Zm00001d027962* (*ZmVPS37A*), *Zm00001d015102* (*ZmGOS13*), and *Zm00001d011347* (*ZmATG7*)], and three for ear and seed development [*Zm00001d011984* (*ZmATG6b*), *Zm00001d042215* (*ZmATG18e*), and *Zm00001d009150* (*ZmSec23a*)], and one for leaf senescence [*Zm00001d042215* (*ZmATG18e*)].

### *ZmSec23a* Is a Maize Improvement Gene Involved in Seed Development

**FIGURE 6:**
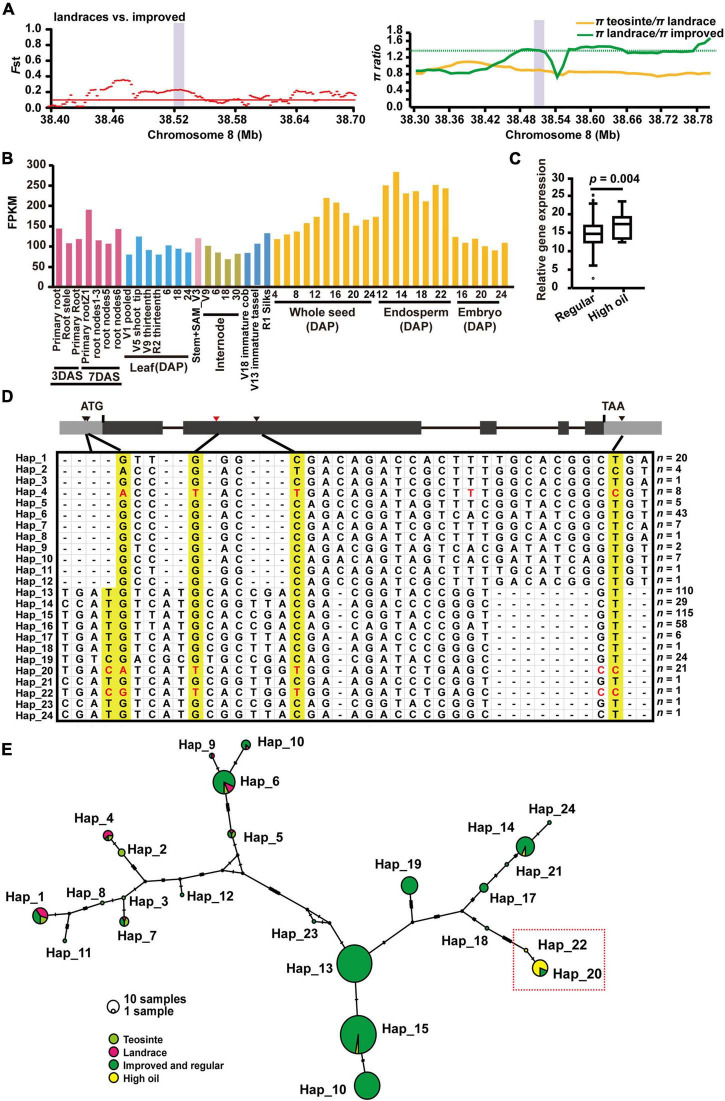
*ZmSec23a* is selected during maize improvement and is strongly associated with seed oil content. **(A)** *Fst* (left) and π (right) values in landraces and improved cultivators across the 0.3-Mb and 0.48-Mb genomic regions surrounding *ZmSec23a*, respectively, which is indicated by the purple bar. **(B)** The spatiotemporal expression profile of *ZmSec23a.* **(C)** Gene expression analysis of *ZmSec23a* in high oil lines and regular materials. Statistical differences between regular materials and high oil lines were calculated by a two-tailed Student’s *t*-test (*p* < 0.05). For the box, the central line indicates the median, and the box bounds mean the 25th and 75th percentiles. **(D)** Haplotype analysis of *ZmSec23a* among teosintes (20), landraces (22), improved lines (403), and high oil lines (23). Yellow colors denote the selected alleles of the *ZmSec23a* gene in maize improved high oil germplasms. **(E)** Genetic relatedness of *ZmSec23a* haplotypes based on the median-joining network constructed using PopART version 1.7. Each haplotype is represented by a circle, with its size proportional to the number of maize lines within it. Light green, teosintes; magenta, landraces; dark green, improved and regular cultivars; yellow, high oil materials.

Among 114 genes involved in seed development, *ZmSec23a* on chromosome 8 was also found to have strong selection signals with high-level population differentiation between landrace and improved lines (*Fst* = 0.211, π landrace/π improved = 1.721) (Figure 6A and Table 1). Given that ZmSec23a is an important COPII component that might regulate the protein biosynthesis during seed development, we first analyzed its mRNA levels using previously reported RNA-sequencing data (Stelpflug et al., 2016). As shown in Figure 6B, *ZmSec23a* showed elevated expression in the endosperm and seed tissues. Interestingly, we also found that *ZmSec23a* exhibited increased mRNA abundance in high oil lines compared with regular materials (Figure 6C).

To gain deeper insights into the evolution of *ZmSec23a*, we then performed haplotype analysis with 468 selected teosintes (20), landraces (22), improved regular lines (403), and high oil lines (23) and identified 24 different haplotypes in its coding region. Among these haplotypes, Hap_20 and Hap_22 are enriched with high oil lines (Figure 6D), with 18 out of 22 being high oil lines. These two haplotypes not only carry the lead SNP (chr8: 38521846; G/T allele) detected by a previous genome association study (Li et al., 2013) but also carry the G/A, C/T, and T/C variants. Interestingly, these variants were also found in Hap_4, which mainly consists of teosintes and landraces, but not modern cultivars (Figure 6D). However, the haplotype network analysis failed to detect the close relationship between Hap_4 and Hap_20/22 (Figure 6E). Moreover, several alleles (Chr8: 38526804, T/C; Chr8: 38526831, G/A; Chr8:38528117, T/C; and Chr8: 38530903, C/T) within the gene region of *ZmSec23a* have different degrees of linkage disequilibrium with the lead SNP (G/T allele) from domestication to improvement, or from regular inbred lines to high oil materials (Supplementary Figure 20), indicating that these alleles of *Zmsec23a* could also be associated with high oil content.

### *ZmVPS37A* Is a Domestication Gene Involved in Pathogen Defense

**FIGURE 7:**
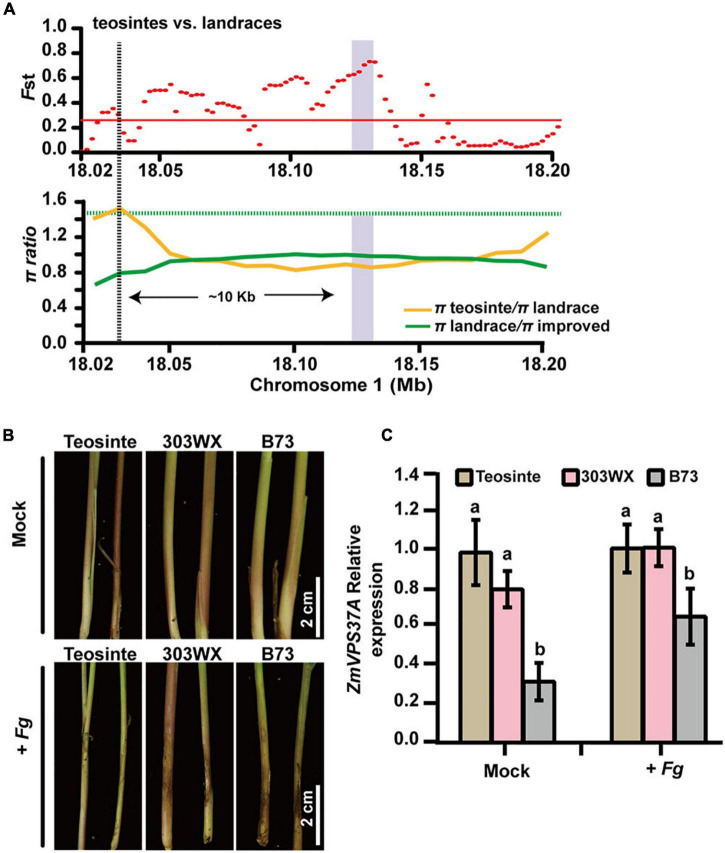
*ZmVPS37A* is a domestication gene involved in pathogen defense. **(A)** *Fst* (upper) and π (lower) values in teosinte and landrace across the 0.18-Mb genomic region surrounding the *ZmVPS37A* gene, which is indicated by the purple bar. **(B)** The stalk phenotypes of teosinte, 303WX, and B73 seedlings 7 days after inoculation by *Fusarium graminearum* PH-1. **(C)** Gene expression analysis of *ZmVPS37A* responding to *F. graminearum* PH-1 48 h after infection. Values are mean ± s.d. Significant differences are shown by different letters (*p* < 0.05).

Another noticeable selected gene is *ZmVPS37A*. As a component of ESCRT machinery, *Arabidopsis* VPS37-1 is required for the endocytosis of plant immune receptor FLS2 after flagellin elicitation and for flg22-triggered stomatal closure to prevent bacterial entrance (Spallek et al., 2013). Moreover, *ZmVPS37A* is significantly associated with variation in Rp1-D21-induced HR (Olukolu et al., 2014). In this study, we found that the genetic diversity of *ZmVPS37A* in landrace was significantly lower than that in teosinte (*Fst* = 0.589, π landrace/π teosinte = 2.154, Figures 5, 7A and Table 1), indicating that it is a domestication gene. To understand why *ZmVPS37A* is selected, we infected the stem tissues of teosinte (W71-2), landrace (303WX), and B73 seedlings with *F. graminearum*. As shown in Figure 7B, teosinte exhibited better resistance than 303WX and B73, while B73 displayed obvious lesions and severe stalk rot symptoms. qRT-PCR analysis revealed that the transcript level of *ZmVPS37A* in teosinte and 303WX was about 1.54-fold compared to B73 control after *F. graminearum* infection (Figure 7C). Analysis of the *cis*-regulatory elements in the promoter regions of *ZmVPS37A* further identified more stress-related elements in teosinte than in B73 (Supplementary Figure 21). These observations suggest that *ZmVPS37A*, like its *Arabidopsis* homolog, is critical for pathogen defense and likely lost its defense function during domestication as a trade-off between plant growth and defense.

## Discussion

As a fundamental cellular process, membrane trafficking in eukaryotic cells is sophisticatedly regulated in response to developmental signals and environmental cues. Previous research has reported that a set of SNARE genes, including *PEN1 (SYP121)*, *VAMP721/722*, and *SNAP33/34*, comprise a conserved regulon together with other ∼50 genes and function as a module in plant innate immunity (Humphry et al., 2010). Moreover, studies on plant autophagy have also revealed the coordinated expression pattern of *ATG* genes, as numerous *ATG* genes are upregulated in senescing tissues, seeds, and under nutrient-limiting conditions (Li et al., 2015; Marshall and Vierstra, 2018). Here, our in-depth RNA-seq analysis showed that genes encoding the same SNARE protein complex have similar expression patterns, implying that some regulons might exist to coordinate their expressions (Figure 2). Our RNA-seq analysis also revealed the tissue specificities of SNARE complexes, indicating the subfunctionalization of expression patterns, and possibly functions too. For example, PM-localized SYP12 syntaxins assemble with other SNAREs, including TGN-localized VAMP72 and SNAP3, into a SNARE complex to mediate the vesicle fusion at the PM. The genes encoding the components of such a SNARE complex could be classified into four groups based on their expression patterns. However, one group (ZmVAMP722/726, ZmNPSN13/14, ZmSYP41, and ZmTYN12) appears to have a constitutive expression pattern, and others show strong root, endosperm, or pollen specificities. During pollen development and pollen tube growth, endomembrane trafficking is one of the essential processes to sustain the growth-promoting membrane dynamics during the reproductive process. ZmVAMP712/728/729, ZmSYP123/125, and ZmSNAP33 compose a pollen-specific group, implying their roles in pollen development. Clearly, the identification of the transcription factors that regulate SNARE genes is required for a full appreciation of the importance of their contributions to plant growth.

Our transcriptomic analysis also revealed that the transcript levels of 114 and 34 membrane-trafficking genes are upregulated in early and late endosperm tissues, respectively (Supplementary Table 1, modules turquoise and yellow, respectively). During seed maturation, the endosperm undergoes three major changes: coenocyte, cellularization, and differentiation (Dai et al., 2021). The involvement of the endomembrane system during endosperm cellularization has been revealed recently by a high temporal-resolution RNA-seq analysis, which shows that multiple membrane-trafficking genes are upregulated at the cellularization stage (Yi et al., 2019). Our study also showed that the genes encoding multiple COPII components, Rab GTPases, and one SNARE are grouped together with those involved in protein assembly and biogenesis by GO enrichment analysis (Figure 3), indicating that these membrane-trafficking components might be essential for protein transport for the cellularization of the endosperm.

The ESCRT is conserved machinery responsible for the formation of intraluminal vesicles (ILVs) to degrade the monoubiquitinated membrane proteins (Gao et al., 2017). Previous studies with rice *OsVPS22* and maize *ZmSAL1* have demonstrated that ESCRT components are essential for seed development (Shen et al., 2003; Zhang et al., 2013). Recent research in barely endosperm has shown that the transcript abundance of three ESCRT genes, namely, *HvTOL1*, *HvTOL2*, and *HvTOL8*, is upregulated in endosperm upon pollination, suggesting their potential roles in vacuolar transport of cargo during early endosperm development (Roustan et al., 2020). In maize, *ZmSKD1* is significantly upregulated under salt and drought conditions as well as in immature seeds (Xia et al., 2013). In this study, our WGCNA analysis revealed that several ESCRT components are involved in the response to abiotic stress (Figure 4 and Supplementary Figure 19). For example, *TOL7* (ESCRT-0 component) and *ISTL6* (SKD1 complex) were found to be upregulated upon fungi infection and were strongly correlated with defense genes and other metabolic processes involved in SA biosynthesis and jasmonic acid signaling pathways (Figure 4 and Supplementary Figure 19), further confirming the important role of ESCRT machinery in maize development and growth.

Many components of the membrane-trafficking system with similar or divergent functions are the consequences of gene duplication, expansion, and neofunctionalization. The proven functionally important differences in the membrane-trafficking system are often accompanied by the emergence of new paralogs of trafficking protein families and/or gene neofunctionalization (More et al., 2020). Therefore, exploring the diversity and the selection of membrane-trafficking genes during maize domestication and improvement was chosen as the subject of this study. The previous genomic analysis of maize genetic diversity has identified the number of genes with strong selection signals during maize evolution from teosinte to modern cultivar by resequencing 73 maize accession genomes (Hufford et al., 2012). Among these genes, a total of 15 and 16 vesicle components are found in domestication and improvement regions, respectively (Hufford et al., 2012). In this study, comparative genomic analysis of teosintes, landraces, and cultivators was conducted renewedly to identify artificially selected vesicle components based on genetic variation diversity. Interestingly, we obtained additional 25 and 14 membrane-trafficking genes, which were located in domestication and improvement regions, respectively (Figure 3). In the maize genome, nine copies of *ZmSec23* were present, and they displayed different spatiotemporal expression patterns, indicating their diverse functions (Supplementary Figure 12). One of them, *ZmSec23a*, was identified as an improvement gene critical for oil production during seed development (Figure 6). Interestingly, the functional diversity of *Sec23* paralogs has been reported previously in *Arabidopsis* by Chung et al. (2016). AtSec23a, the homolog of ZmSec23a, was found to interact specifically with AtSar1a and be upregulated under ER stress (Zeng et al., 2015; Chung et al., 2016). A unique amino acid, Cys484, in AtSec23a required for the interaction with AtSar1a was found to be conserved in ZmSec23a (Cys470) (data not shown). Therefore, whether any maize Sar1 isoform interacts with ZmSec23a to participate in seed development will be an interesting avenue for future research.

Among the selected genes, seven are for the autophagy pathway, of which four of them have been already identified by QTL mapping or associated studies (Supplementary Table 4). For example, *ZmATG7* was found within selected improvement regions in our study and has also been identified as one of the targeted genomic regions during modern maize breeding in a genome-wide association study with 350 inbred lines (Wang et al., 2020a). A non-synonymous variant (Chr8_142693004, G-A, and S662L) in *ZmATG7* shows significant changes in allele frequency across the breeding eras of modern maize, suggesting *ZmATG7* is a particularly interesting candidate for the selection for biotic resistance. Moreover, *ZmATG18e* (*Zm00001d042215*) has been identified as a novel locus for leaf senescence regulation, with lower *ZmATG18e* expression significantly delaying leaf senescence (Feng et al., 2021). All these studies suggested the important role(s) of autophagy in maize development and stress resistance.

## Data Availability Statement

The datasets presented in this study can be found in online repositories. The names of the repository/repositories and accession number(s) can be found below: The data used for WGCNA analysis were derived from the GEO and NCBI database. The RNA-seq data of maize B73 different tissues/stages have been reported previously (Stelpflug et al., 2016) and were deposited in SAR (accession numbers PRJNA171684 and SRP010680). The Illumina HiSeq data of Gibberella ear rot disease caused by *Fusarium graminearum* have been deposited in GEO (GSE92448) and SAR (accession numbers PRJNA357594 and SRP095179) (Kebede et al., 2018).

## Author Contributions

FL designed the research. CZ and YY conducted the experiments. CZ, YY, GD, and HL performed the data analysis. FL and CZ wrote the manuscript. All authors contributed to the article and approved the submitted version.

## Conflict of Interest

The authors declare that the research was conducted in the absence of any commercial or financial relationships that could be construed as a potential conflict of interest.

## Publisher’s Note

All claims expressed in this article are solely those of the authors and do not necessarily represent those of their affiliated organizations, or those of the publisher, the editors and the reviewers. Any product that may be evaluated in this article, or claim that may be made by its manufacturer, is not guaranteed or endorsed by the publisher.

## Footnotes

1
http://plants.ensembl.org/index.html

2
http://www.arabidopsis.org/

3
http://bioinformatics.cau.edu.cn/MCENet/index.php

4
http://pfam.sanger.ac.uk/

5
https://www.evolgenius.info

6
https://www.ncbi.nlm.nih.gov/Structure/bwrpsb/bwrpsb.cgi

7
https://gephi.org/users/download/

8
www.maizego.org

9
http://www.R-project.org

10
https://cbsusrv04.tc.cornell.edu/users/panzea/download.aspx?Filegroupid=15

11
http://maizego.org/Resources.html

12
http://www.maizego.org

13
http://bioinformatics.psb.ugent.be/webtools/plantcare/html/

14
https://plants.ensembl.org/Zea_mays/Info/Annotation/

15
https://www.ncbi.nlm.nih.gov/Structure/cdd/cdd.shtml

16
http://pfam.xfam.org/

17
https://maizegdb.org/

## References

[B1] Barrett J. C. Fry B. Maller J. Daly M. J. (2005). Haploview: analysis and visualization of LD and haplotype maps. *Bioinformatics* 21 263–265. 10.1093/bioinformatics/bth457 15297300

[B2] Bassham D. C. Brandizzi F. Otegui M. S. Sanderfoot A. A. (2008). The secretory system of *Arabidopsis*. *Arabidopsis Book* 6:e0116. 10.1199/tab.0116 22303241 PMC3243370

[B3] Bhandari D. D. Brandizzi F. (2020). Plant endomembranes and cytoskeleton: moving targets in immunity. *Curr. Opin. Plant Biol.* 58 8–16. 10.1016/j.pbi.2020.09.003 33099211

[B4] Bradbury P. J. Zhang Z. Kroon D. E. Casstevens T. M. Ramdoss Y. Buckler E. S. (2007). TASSEL: software for association mapping of complex traits in diverse samples. *Bioinformatics* 23 2633–2635. 10.1093/bioinformatics/btm308 17586829

[B5] Bukowski R. Guo X. Lu Y. Zou C. He B. Rong Z. (2018). Construction of the third-generation *Zea mays* haplotype map. *Gigascience* 7 1–12. 10.1093/gigascience/gix134 29300887 PMC5890452

[B6] Chen L. Li Y. X. Li C. H. Shi Y. S. Song Y. C. Zhang D. F. (2020b). The retromer protein ZmVPS29 regulates maize kernel morphology likely through an auxin-dependent process(es). *Plant Biotechnol. J.* 18 1004–1014. 10.1111/pbi.13267 31553822 PMC7061865

[B7] Chen C. J. Chen H. Zhang Y. Thomas H. R. Frank M. H. He Y. H. (2020a). TBtools: an integrative toolkit developed for interactive analyses of big biological data. *Mol. Plant* 13 1194–1202. 10.1016/j.molp.2020.06.009 32585190

[B8] Chung K. B. Zeng Y. L. Li Y. M. Ji C. Y. Xia Y. J. Jiang L. W. (2018). Signal motif-dependent ER export of the Qc-SNARE BET12 interacts with MEMB12 and affects PR1 trafficking in *Arabidopsis*. *J. Cell Sci*. 131:jcs202838. 10.1242/jcs.202838 28546447

[B9] Chung K. P. Zeng Y. L. Jiang L. W. (2016). COPII paralogs in plants: functional redundancy or diversity? *Trends Plant Sci.* 21 758–769. 10.1016/j.tplants.2016.05.010 27317568

[B10] Collins N. C. Thordal-Christensen H. Lipka V. Bau S. Kombrink E. Qiu J. L. (2003). SNARE-protein-mediated disease resistance at the plant cell wall. *Nature* 425 973–977. 10.1038/nature02076 14586469

[B11] Dai D. W. Ma Z. Y. Song R. T. (2021). Maize endosperm development. *J. Integr. Plant Biol.* 63 613–627. 10.1111/jipb.13069 33448626

[B12] Danecek P. Auton A. Abecasis G. Albers C. A. Banks E. DePristo M. A. (2011). The variant call format and VCFtools. *Bioinformatics* 27 2156–2158. 10.1093/bioinformatics/btr330 21653522 PMC3137218

[B13] Feng X. Liu L. L. Li Z. G. Sun F. Wu X. Y. Hao D. Y. (2021). Potential interaction between autophagy and auxin during maize leaf senescence. *J. Exp. Bot.* 72 3554–3568. 10.1093/jxb/erab094 33684202 PMC8446287

[B14] Fu J. J. Cheng Y. B. Linghu J. J. Yang X. H. Kang L. K. Zhang Z. X. (2013). RNA sequencing reveals the complex regulatory network in the maize kernel. *Nat. Commun.* 4:2832. 10.1038/ncomms3832 24343161

[B15] Gamir J. Pastor V. Sanchez-Bel P. Agut B. Mateu D. Garcia-Andrade J. (2018). Starch degradation, abscisic acid and vesicular trafficking are important elements in callose priming by indole-3-carboxylic acid in response to *Plectosphaerella cucumerina* infection. *Plant J.* 96 518–531. 10.1111/tpj.14045 30051514

[B16] Gao C. J. Zhuang X. Shen J. B. Jiang L. W. (2017). Plant ESCRT Complexes: moving beyond endosomal sorting. *Trends Plant Sci.* 22 986–998. 10.1016/j.tplants.2017.08.003 28867368

[B17] Gu Y. N. Zavaliev R. Dong X. N. (2017). Membrane trafficking in plant immunity. *Mol. Plant* 10 1026–1034. 10.1016/j.molp.2017.07.001 28698057 PMC5673114

[B18] He M. Lan M. Zhang B. C. Zhou Y. Y. Wang Y. Q. Zhu L. (2018). Rab-H1b is essential for trafficking of cellulose synthase and for hypocotyl growth in *Arabidopsis thaliana*. *J. Integr. Plant Biol.* 60 1051–1069. 10.1111/jipb.12694 29975455

[B19] He W. Wang L. Lin Q. L. Yu F. (2021). Rice seed storage proteins: biosynthetic pathways and the effects of environmental factors. *J. Integr. Plant Biol.* 63 1999–2019. 10.1111/jipb.13176 34581486

[B20] Heucken N. Ivanov R. (2018). The retromer, sorting nexins and the plant endomembrane protein trafficking. *J. Cell Sci.* 131:jcs203695. 10.1242/jcs.203695 29061884

[B21] Hofius D. Li L. Hafren A. Coll N. S. (2017). Autophagy as an emerging arena for plant-pathogen interactions. *Curr. Opin. Plant Biol.* 38 117–123. 10.1016/j.pbi.2017.04.017 28545004

[B22] Hufford M. B. Xu X. van Heerwaarden J. Pyhajarvi T. Chia J. M. Cartwright R. A. (2012). Comparative population genomics of maize domestication and improvement. *Nat. Genet.* 44 808–811. 10.1038/ng.2309 22660546 PMC5531767

[B23] Humphry M. Bednarek P. Kemmerling B. Koh S. Stein M. Gobel U. (2010). A regulon conserved in monocot and dicot plants defines a functional module in antifungal plant immunity. *Proc. Natl. Acad. Sci. U. S. A.* 107 21896–21901. 10.1073/pnas.1003619107 21098265 PMC3003077

[B24] Inada N. Ueda T. (2014). Membrane trafficking pathways and their roles in plant-microbe interactions. *Plant Cell Physiol.* 55 672–686. 10.1093/pcp/pcu046 24616268

[B25] Jiao Y. Zhao H. N. Ren L. H. Song W. B. Zeng B. Guo J. J. (2012). Genome-wide genetic changes during modern breeding of maize. *Nat. Genet.* 44 812–815. 10.1038/ng.2312 22660547

[B26] Kalde M. Nuhse T. S. Findlay K. Peck S. C. (2007). The syntaxin SYP132 contributes to plant resistance against bacteria and secretion of pathogenesis-related protein 1. *Proc. Natl. Acad. Sci. U. S. A.* 104 11850–11855. 10.1073/pnas.0701083104 17592123 PMC1913864

[B27] Kebede A. Z. Johnston A. Schneiderman D. Bosnich W. Harris L. J. (2018). Transcriptome profiling of two maize inbreds with distinct responses to Gibberella ear rot disease to identify candidate resistance genes. *BMC Genomics* 19:131. 10.1186/s12864-018-4513-4 29426290 PMC5807830

[B28] Kwon C. Neu C. Pajonk S. Yun H. S. Lipka U. Humphry M. (2008). Co-option of a default secretory pathway for plant immune responses. *Nature* 451 835–840. 10.1038/nature06545 18273019

[B29] Langfelder P. Horvath S. (2008). WGCNA: an R package for weighted correlation network analysis. *BMC Bioinformatics* 9:559. 10.1186/1471-2105-9-559 19114008 PMC2631488

[B30] Leigh J. W. Bryant D. (2015). POPART: full-feature software for haplotype network construction. *Methods Ecol. Evol.* 6 1110–1116. 10.1111/2041-210X.12410

[B31] Li F. Q. Chung T. Pennington J. G. Federico M. L. Kaeppler H. F. Kaeppler S. M. (2015). Autophagic recycling plays a central role in maize nitrogen remobilization. *Plant Cell* 27 1389–1408. 10.1105/tpc.15.00158 25944100 PMC4456646

[B32] Li H. Peng Z. Y. Yang X. H. Wang W. D. Fu J. J. Wang J. H. (2013). Genome-wide association study dissects the genetic architecture of oil biosynthesis in maize kernels. *Nat. Genet.* 45 43–50. 10.1038/ng.2484 23242369

[B33] Librado P. Rozas J. (2009). DnaSP v5: a software for comprehensive analysis of DNA polymorphism data. *Bioinformatics* 25 1451–1452. 10.1093/bioinformatics/btp187 19346325

[B34] Lin T. Zhu G. T. Zhang J. Xu X. H. Yu Q. H. Zheng Z. (2014). Genomic analyses provide insights into the history of tomato breeding. *Nat. Genet.* 46 1220–1226. 10.1038/ng.3117 25305757

[B35] Liu H. J. Wang X. Q. Warburton M. L. Wen W. W. Jin M. L. Deng M. (2015). Genomic, transcriptomic, and phenomic variation reveals the complex adaptation of modern maize breeding. *Mol. Plant* 8 871–884. 10.1016/j.molp.2015.01.016 25620769

[B36] Lu S. N. Wang J. Y. Chitsaz F. Derbyshire M. K. Geer R. C. Gonzales N. R. (2020). CDD/SPARCLE: the conserved domain database in 2020. *Nucleic Acids Res.* 48 D265–D268. 10.1093/nar/gkz991 31777944 PMC6943070

[B37] Marshall R. S. Vierstra R. D. (2018). Autophagy: the master of bulk and selective recycling. *Annu. Rev. Plant Biol.* 29 173–208. 10.1146/annurev-arplant-042817-040606 29539270

[B38] Minamino N. Ueda T. (2019). RAB GTPases and their effectors in plant endosomal transport. *Curr. Opin. Plant Biol.* 52 61–68. 10.1016/j.pbi.2019.07.007 31454706

[B39] More K. Klinger C. M. Barlow L. D. Dacks J. B. (2020). Evolution and natural history of membrane trafficking in eukaryotes. *Curr. Biol.* 30 R553–R564. 10.1016/j.cub.2020.03.068 32428497

[B40] Olukolu B. A. Wang G. F. Vontimitta V. Venkata B. P. Marla S. Ji J. (2014). A genome-wide association study of the maize hypersensitive defense response identifies genes that cluster in related pathways. *PLoS Genet.* 10:e1004562. 10.1371/journal.pgen.1004562 25166276 PMC4148229

[B41] Paez Valencia J. Goodman K. Otegui M. S. (2016). Endocytosis and endosomal trafficking in plants. *Annu. Rev. Plant Biol.* 67 309–335. 10.1146/annurev-arplant-043015-112242 27128466

[B42] Paul P. Simm S. Mirus O. Scharf K. D. Fragkostefanakis S. Schleiff E. (2014). The complexity of vesicle transport factors in plants examined by orthology search. *PLoS One* 9:e97745. 10.1371/journal.pone.0097745 24844592 PMC4028247

[B43] Rodriguez-Furlan C. Minina E. A. Hicks G. R. (2019). Remove, recycle, degrade: regulating plasma membrane protein accumulation. *Plant Cell* 31 2833–2854. 10.1105/tpc.19.00433 31628169 PMC6925004

[B44] Roustan V. Hilscher J. Weidinger M. Reipert S. Shabrangy A. Gebert C. (2020). Protein sorting into protein bodies during barley endosperm development is putatively regulated by cytoskeleton members, MVBs and the HvSNF7s. *Sci. Rep.* 10:1864. 10.1038/s41598-020-58740-x 32024857 PMC7002727

[B45] Shen B. Li C. Min Z. Meeley R. B. Tarczynski M. C. Olsen O. A. (2003). *sal1* determines the number of aleurone cell layers in maize endosperm and encodes a class E vacuolar sorting protein. *Proc. Natl. Acad. Sci. U. S. A.* 100 6552–6557. 10.1073/pnas.0732023100 12750475 PMC164484

[B46] Spallek T. Beck M. Ben Khaled S. Salomon S. Bourdais G. Schellmann S. (2013). ESCRT-I mediates FLS2 endosomal sorting and plant immunity. *PLoS Genet.* 9:e1004035. 10.1371/journal.pgen.1004035 24385929 PMC3873229

[B47] Speth E. B. Imboden L. Hauck P. He S. Y. (2009). Subcellular localization and functional analysis of the *Arabidopsis* GTPase RabE. *Plant Physiol.* 149 1824–1837. 10.1104/pp.108.132092 19233904 PMC2663744

[B48] Stelpflug S. C. Sekhon R. S. Vaillancourt B. Hirsch C. N. Buell C. R. de Leon N. (2016). An expanded maize gene expression atlas based on RNA sequencing and its use to explore root development. *Plant Genome* 9 1–16. 10.3835/plantgenome2015.04.0025 27898762

[B49] Surpin M. Raikhel N. (2004). Traffic jams affect plant development and signal transduction. *Nat. Rev. Mol. Cell Biol.* 5 100–109. 10.1038/nrm1311 15040443

[B50] Takacs E. M. Suzuki M. Scanlon M. J. (2012). Discolored1 (DSC1) is an ADP-ribosylation factor-GTPase activating protein required to maintain differentiation of maize kernel structures. *Front. Plant Sci.* 3:115. 10.3389/fpls.2012.00115 22666226 PMC3364507

[B51] Tian T. You Q. Yan H. Y. Xu W. Y. Su Z. (2018). MCENet: a database for maize conditional co-expression network and network characterization collaborated with multi-dimensional omics levels. *J. Genet. Genomics* 45 351–360. 10.1016/j.jgg.2018.05.007 30057343

[B52] Wang B. B. Lin Z. C. Li X. Zhao Y. P. Zhao B. B. Wu G. X. (2020a). Genome-wide selection and genetic improvement during modern maize breeding. *Nat. Genet.* 52 565–571. 10.1038/s41588-020-0616-3 32341525

[B53] Wang X. F. Xu M. Gao C. J. Zeng Y. L. Cui Y. Shen W. J. (2020b). The roles of endomembrane trafficking in plant abiotic stress responses. *J. Integr. Plant Biol.* 62 55–69. 10.1111/jipb.12895 31829507

[B54] Xia Z. L. Wei Y. Y. Sun K. L. Wu J. Y. Wang Y. X. Wu K. (2013). The maize AAA-type protein SKD1 confers enhanced salt and drought stress tolerance in transgenic tobacco by interacting with Lyst-interacting protein 5. *PLoS One* 8:e69787. 10.1371/journal.pone.0069787 23894539 PMC3722157

[B55] Yi F. Gu W. Chen J. Song N. Gao X. Zhang X. B. (2019). High temporal-resolution transcriptome landscape of early maize seed development. *Plant Cell* 31 974–992. 10.1105/tpc.18.00961 30914497 PMC6533015

[B56] Yu G. C. Wang L. G. Han Y. Y. He Q. Y. (2012). clusterProfiler: an R package for comparing biological themes among gene clusters. *OMICS* 16 284–287. 10.1089/omi.2011.0118 22455463 PMC3339379

[B57] Zeng Y. L. Chung K. B. Li B. Y. Lai C. M. Lam S. K. Wang X. F. (2015). Unique COPII component AtSar1a/AtSec23a pair is required for the distinct function of protein ER export in *Arabidopsis thaliana*. *Proc. Natl. Acad. Sci. U. S. A*. 112 14360–14365. 10.1073/pnas.1519333112 26578783 PMC4655569

[B58] Zhang X. Q. Hou P. Zhu H. T. Li G. D. Liu X. G. Xie X. M. (2013). Knockout of the VPS22 component of the ESCRT-II complex in rice (*Oryza sativa* L.) causes chalky endosperm and early seedling lethality. *Mol. Biol. Rep.* 40 3475–3481. 10.1007/s11033-012-2422-1 23275199

[B59] Zheng P. Zheng C. Y. Otegui M. S. Li F. Q. (2022). Endomembrane mediated trafficking of seed storage proteins: from *Arabidopsis* to cereal crops. *J. Exp. Bot.* 73 1312–1326. 10.1093/jxb/erab519 34849750

[B60] Zhou Z. K. Jiang Y. Wang Z. Gou Z. H. Lyu J. Li W. Y. (2015). Resequencing 302 wild and cultivated accessions identifies genes related to domestication and improvement in soybean. *Nat. Biotechnol.* 33 408–414. 10.1038/nbt.3096 25643055

