# Supplementary material for: Network and Evolutionary Analysis Reveals Candidate Genes of Membrane Trafficking Involved in Maize Seed Development and Immune Response
Source: Front Plant Sci. 2022 Jun 24;13:883961. doi: 10.3389/fpls.2022.883961 (PMC9263852; doi:10.3389/fpls.2022.883961)
Supplement: Supplementary file 2 [file Data_Sheet_2.pdf]

Supplementary Figures S1-S21

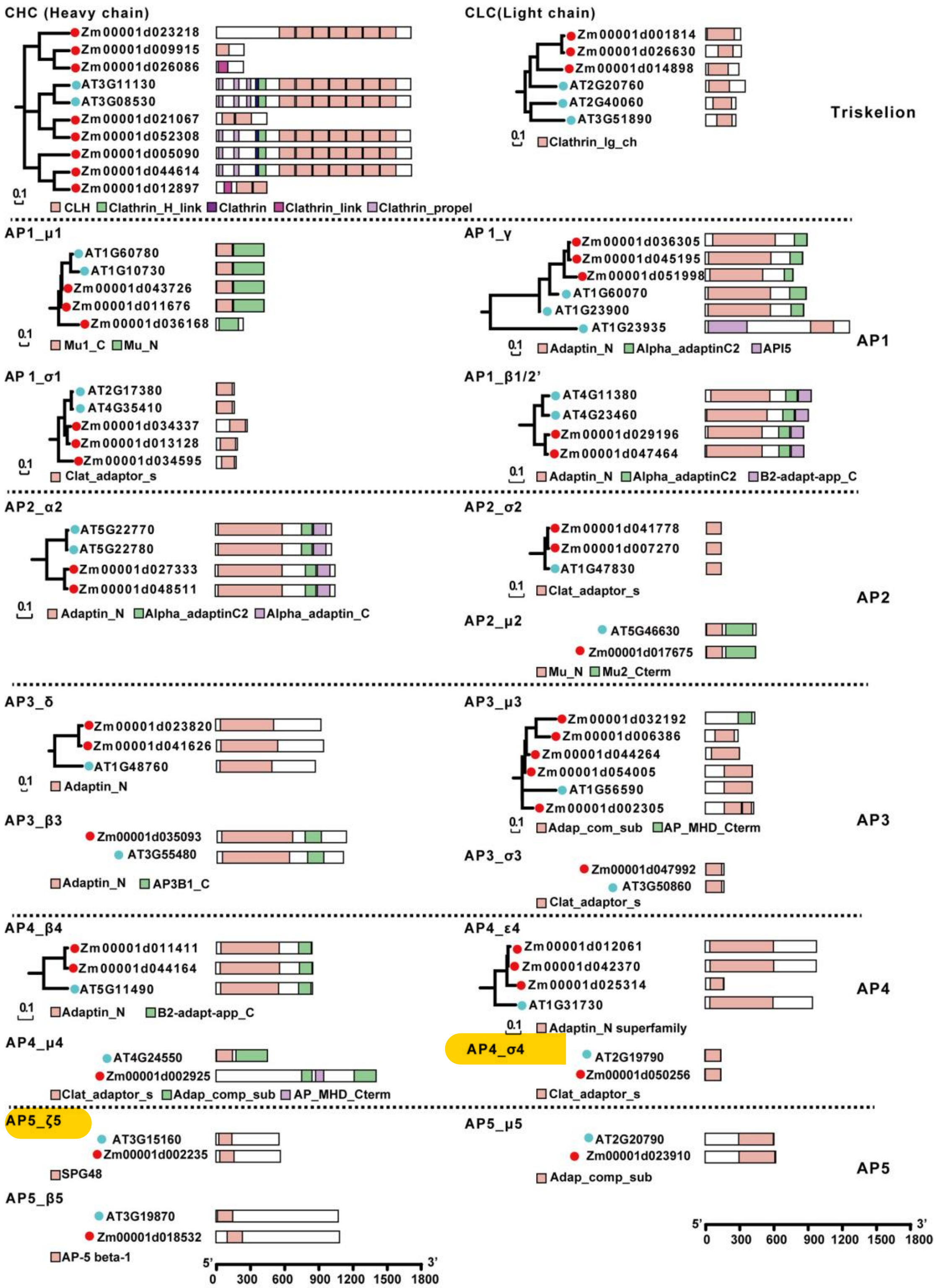

Supplementary Figure S1. Phylogenetic and domain analysis of various components of Clathrin-Coated protein complex and adaptor complexes from *Arabidopsis* and maize. The full-length amino acid sequences of various components were aligned by ClustalW, and phylogenetic trees were created using MEGA (version 6.0) with the Neighbor-Joining method (bootstrap value set at 1,000). Protein domains were visualized by TBtools software (v1.09854). Evolview-v2 software was used for visualization phylogenetic trees. The cyan and red dots indicate protein from *Arabidopsis* and maize, respectively. At, *Arabidopsis thaliana*; Zm, *Zea mays*. Tree

scale: 0.1.

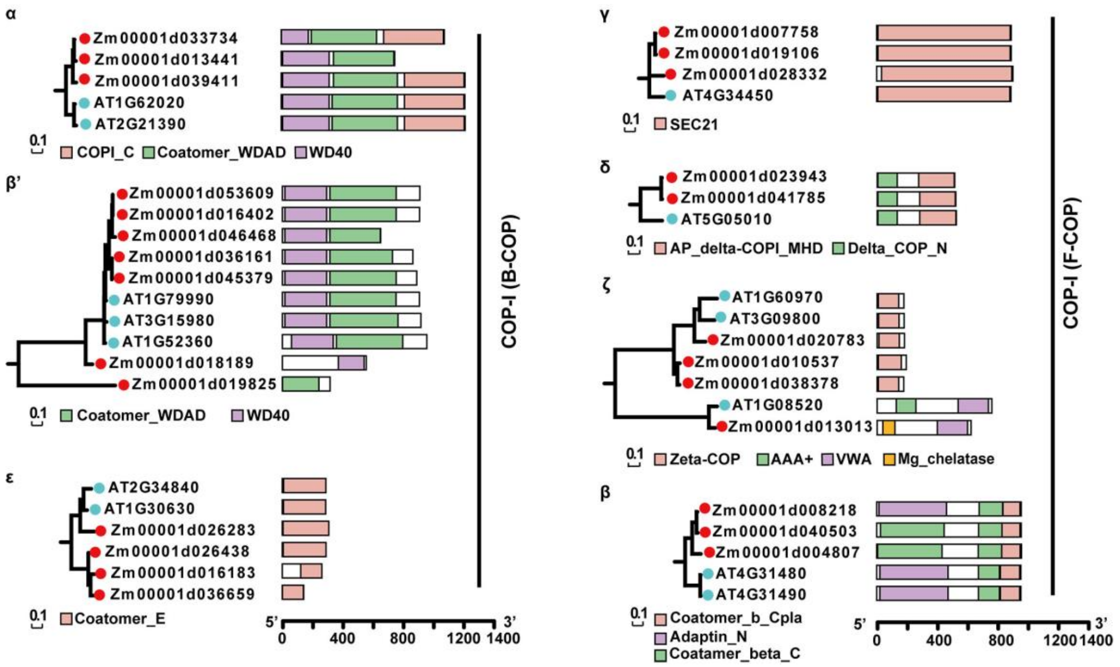

Supplementary Figure S2. Phylogenetic and domain analysis of various components of COP-I complex from *Arabidopsis* and maize. The full-length amino acid sequences of factors were aligned by ClustalW, and phylogenetic trees were created using MEGA (version 6.0) with the Neighbor-Joining method (bootstrap value set at 1,000). Protein domains were visualized by TBtools software (v1.09854). Evolview-v2 software was used for visualization phylogenetic trees. The cyan and red dots indicate protein from *Arabidopsis* and maize, respectively. At, *Arabidopsis thaliana*; Zm, *Zea mays*. Tree scale: 0.1.

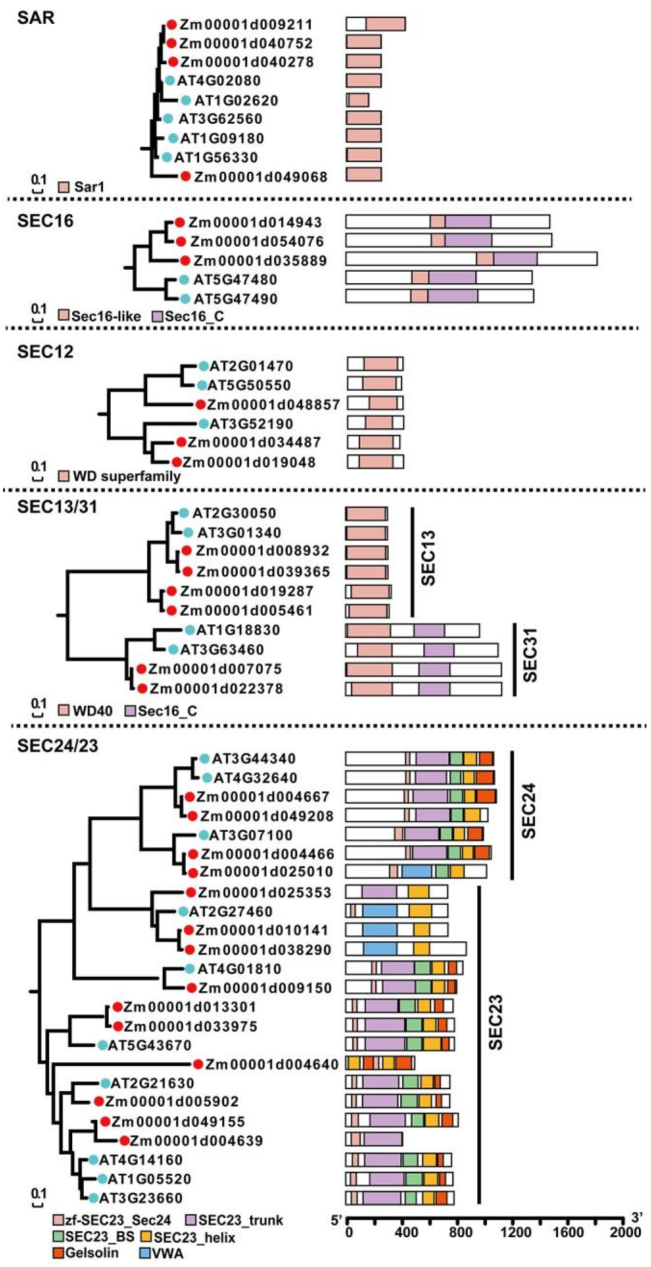

Supplementary Figure S3. Phylogenetic and domain analysis of various components of COP-II complex from *Arabidopsis* and maize. The full-length amino acid sequences of factors were aligned by ClustalW, and phylogenetic trees were created using MEGA (version 6.0) with the Neighbor-Joining method (bootstrap value set at 1,000). Protein domains were visualized by TBtools software (v1.09854). Evolview-v2 software was used for visualization phylogenetic trees. The cyan and red dots indicate protein from *Arabidopsis* and maize, respectively. At, *Arabidopsis thaliana*; Zm, *Zea mays*. Tree scale: 0.1.

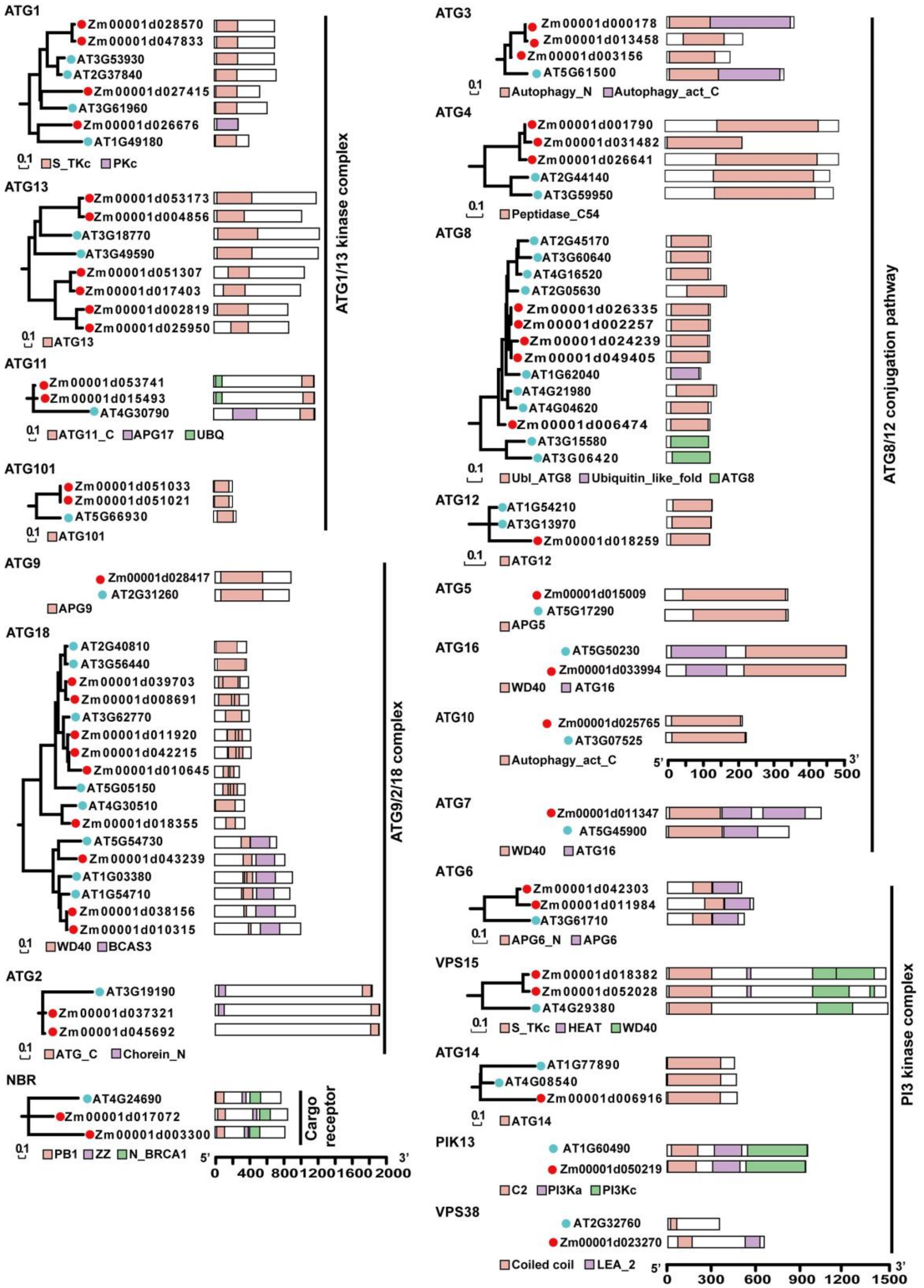

Supplementary Figure S4. Phylogenetic and domain analysis of various components of autophagy machinery from *Arabidopsis* and maize. The full-length amino acid sequences of factors were aligned by ClustalW, and phylogenetic trees were created using MEGA (version 6.0) with the Neighbor-Joining method (bootstrap value set at 1,000). Protein domains were visualized by TBtools software (v1.09854). Evolview-v2 software was used for visualization phylogenetic trees. The cyan and red dots indicate

protein from *Arabidopsis* and maize, respectively. At, *Arabidopsis thaliana*; Zm, *Zea mays*. Tree scale:0.1.

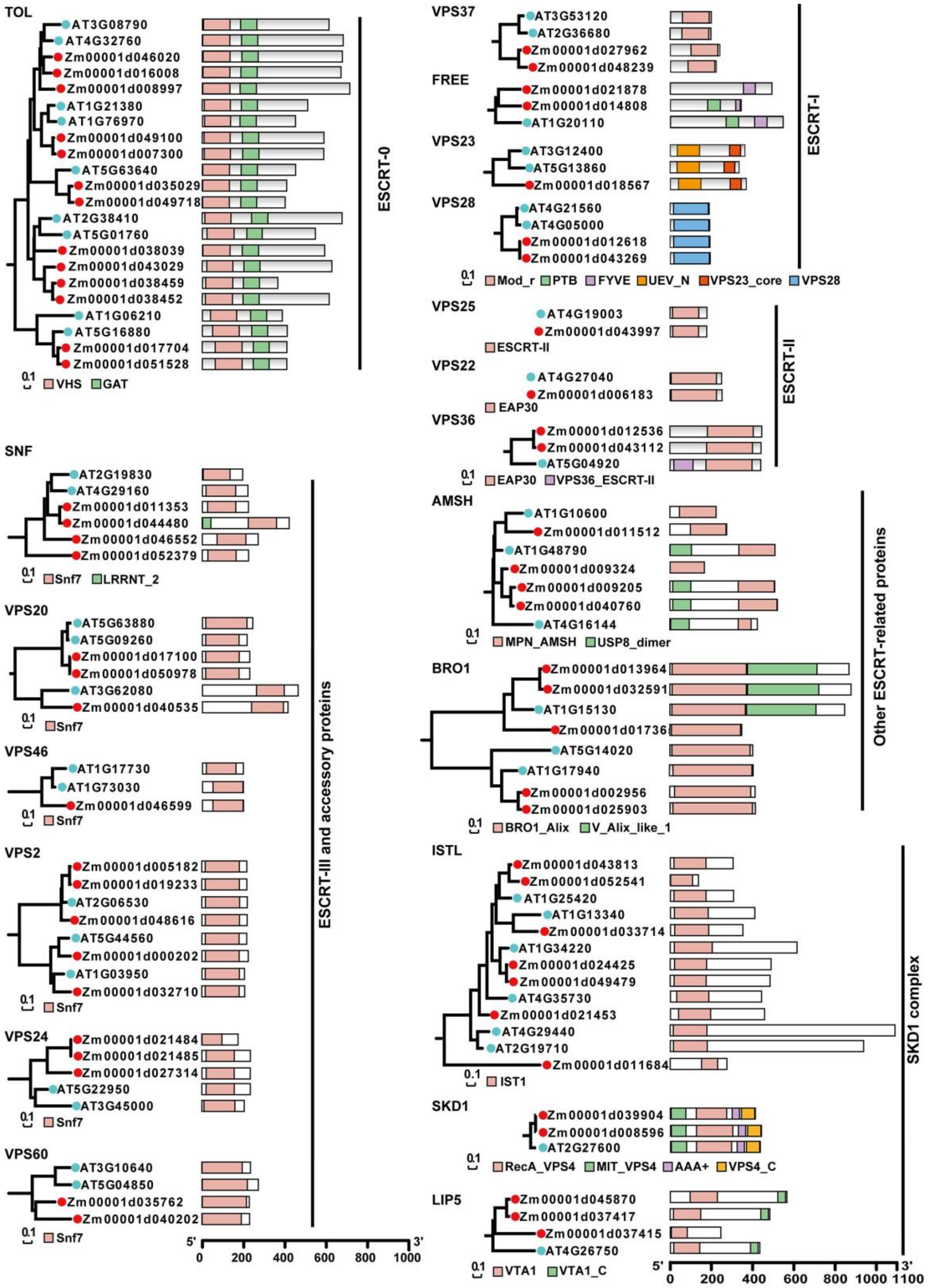

Supplementary Figure S5. Phylogenetic and domain analysis of various components of ESCRT machinery from *Arabidopsis* and maize. The full-length amino acid sequences of factors were aligned by ClustalW, and phylogenetic trees were created using MEGA (version 6.0) with the Neighbor-Joining method (bootstrap value set at 1,000). Protein domains were visualized by TBtools software (v1.09854). Evolview-v2 software was used for visualization phylogenetic trees. The cyan and red dots indicate

protein from *Arabidopsis* and maize, respectively. At, *Arabidopsis thaliana*; Zm, *Zea mays*. Tree scale: 0.1.

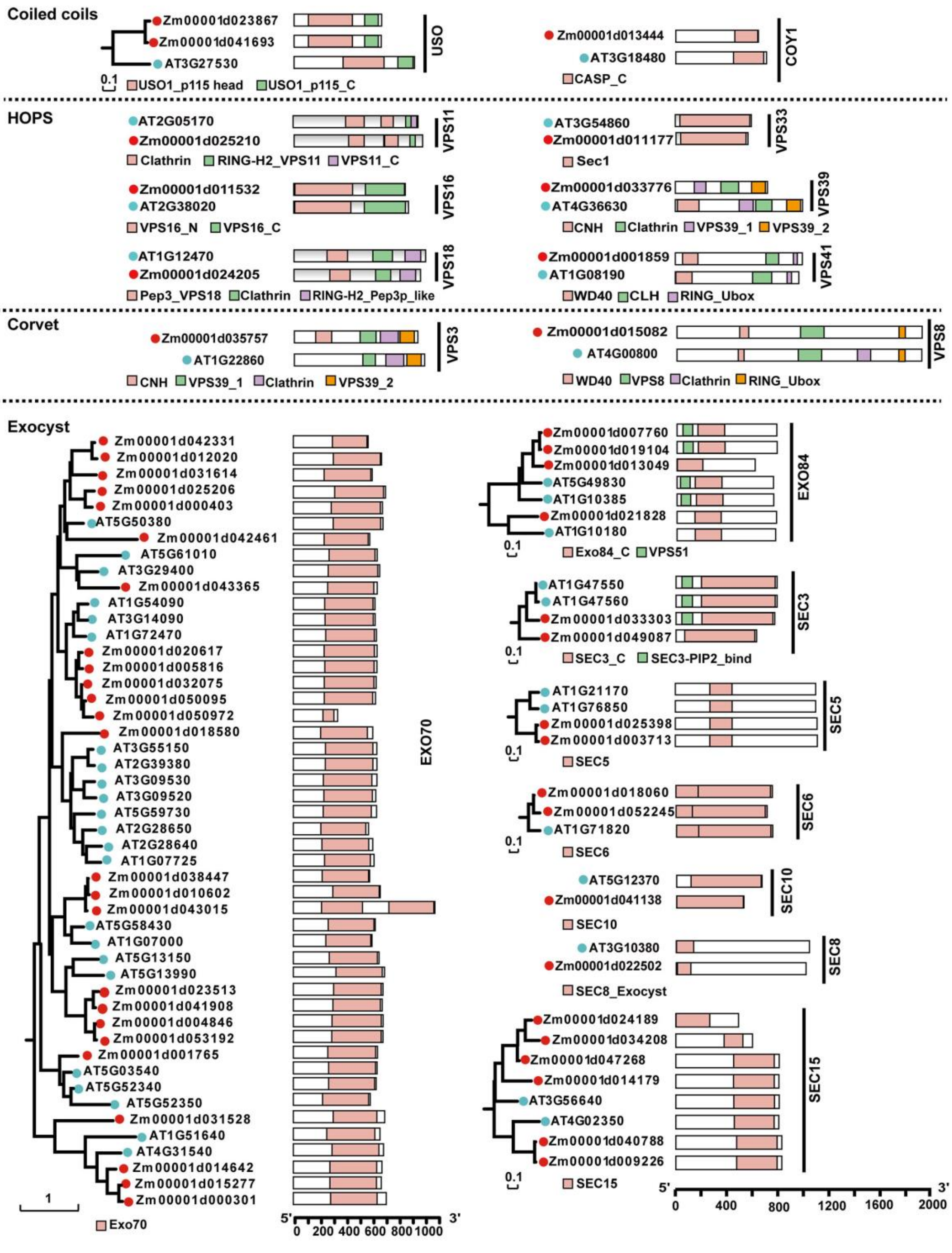

Coiled coils
Zm00001d023867
Zm00001d041693
AT3G27530
USO
0.1
USO1_p115 head
USO1_p115_C
Zm00001d013444
AT3G18480
COY1
CASP_C
HOPS
AT2G05170
Zm00001d025210
VPS11
Clathrin
RING-H2_VPS11
VPS11_C
AT3G54860
Zm00001d011177
VPS33
Sec1
Zm00001d011532
AT2G38020
VPS16
VPS16_N
VPS16_C
Zm00001d033776
AT4G36630
VPS39
CNH
Clathrin
VPS39_1
VPS39_2
AT1G12470
Zm00001d024205
VPS18
Pep3_VPS18
Clathrin
RING-H2_Pep3p_like
Zm00001d001859
AT1G08190
VPS41
WD40
CLH
RING_Ubox
Corvet
Zm00001d035757
AT1G22860
VPS3
CNH
VPS39_1
Clathrin
VPS39_2
Zm00001d015082
AT4G00800
VPS8
WD40
VPS8
Clathrin
RING_Ubox
Exocyst
Zm00001d042331
Zm00001d012020
Zm00001d031614
Zm00001d025206
Zm00001d000403
AT5G50380
Zm00001d042461
AT5G61010
AT3G29400
Zm00001d043365
AT1G54090
AT3G14090
AT1G72470
Zm00001d020617
Zm00001d005816
Zm00001d032075
Zm00001d050095
Zm00001d050972
Zm00001d018580
AT3G55150
AT2G39380
AT3G09530
AT3G09520
AT5G59730
AT2G28650
AT2G28640
AT1G07725
Zm00001d038447
Zm00001d010602
Zm00001d043015
AT5G58430
AT1G07000
AT5G13150
AT5G13990
Zm00001d023513
Zm00001d041908
Zm00001d004846
Zm00001d053192
Zm00001d001765
AT5G03540
AT5G52340
AT5G52350
Zm00001d031528
AT1G51640
AT4G31540
Zm00001d014642
Zm00001d015277
Zm00001d000301
EXO70
1
Exo70
5'
3'
0 200 400 600 800 1000
Zm00001d007760
Zm00001d019104
Zm00001d013049
AT5G49830
AT1G10385
Zm00001d021828
AT1G10180
EXO84
0.1
Exo84_C
VPS51
AT1G47550
AT1G47560
Zm00001d033303
Zm00001d049087
SEC3
0.1
SEC3_C
SEC3-PIP2_bind
AT1G21170
AT1G76850
Zm00001d025398
Zm00001d003713
SEC5
0.1
SEC5
Zm00001d018060
Zm00001d052245
AT1G71820
SEC6
0.1
SEC6
AT5G12370
Zm00001d041138
SEC10
SEC10
AT3G10380
Zm00001d022502
SEC8
SEC8_Exocyst
Zm00001d024189
Zm00001d034208
Zm00001d047268
Zm00001d014179
AT3G56640
AT4G02350
Zm00001d040788
Zm00001d009226
SEC15
0.1
SEC15
5'
3'
0 400 800 1200 1600 2000

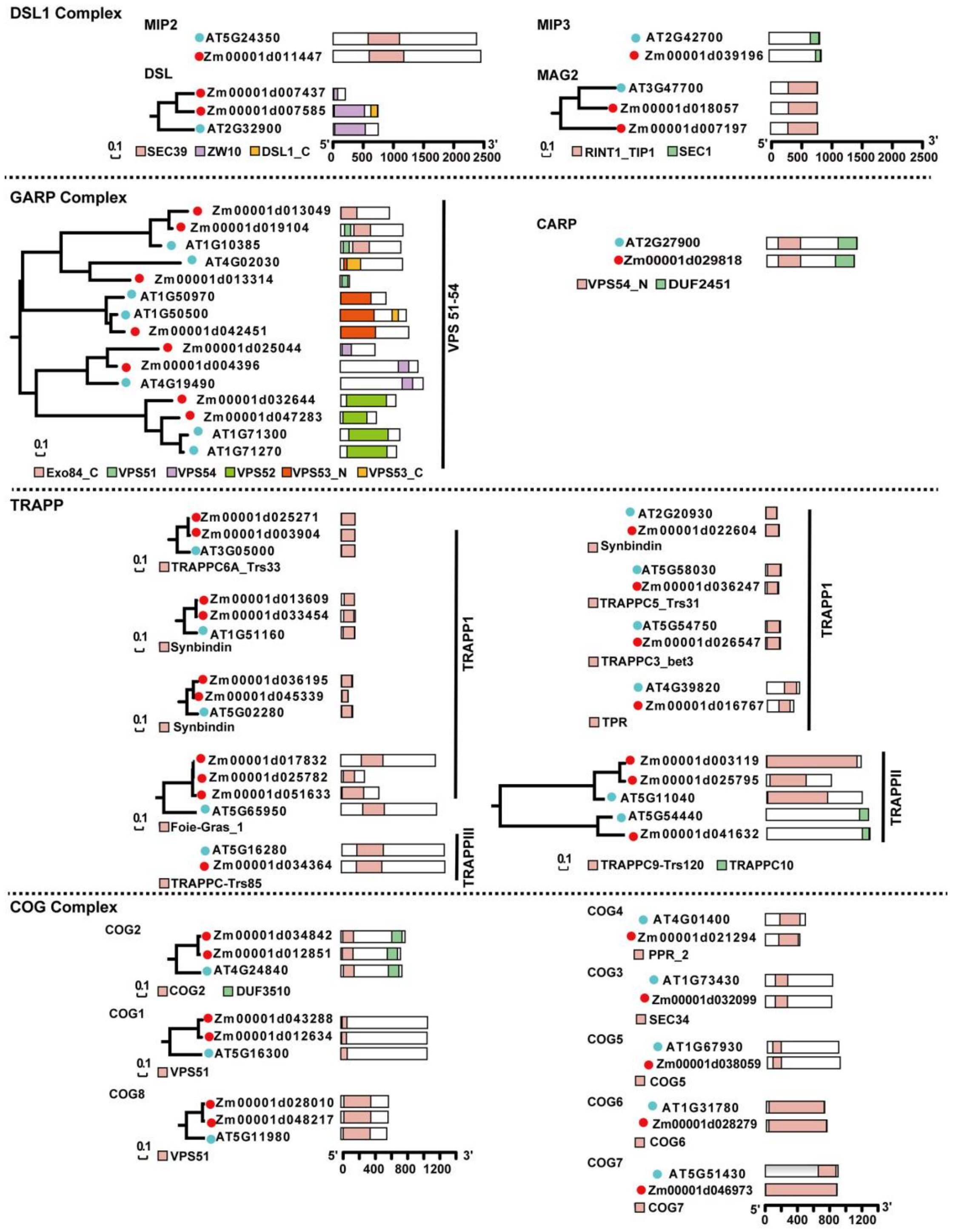

Supplementary Figure S6. Phylogenetic and domain analysis of various components of Tethering complexes from *Arabidopsis* and maize. The full-length amino acid sequences of factors were aligned by ClustalW, and phylogenetic trees were created using MEGA (version 6.0) with the Neighbor-Joining method (bootstrap value set at 1,000). Protein domains were visualized by TBtools software (v1.09854). Evolview-v2 software was used for visualization phylogenetic trees. The cyan and red dots indicate protein from *Arabidopsis* and maize, respectively. At, *Arabidopsis thaliana*; Zm, *Zea mays*. The number 0.1 and 1 were tree scale.

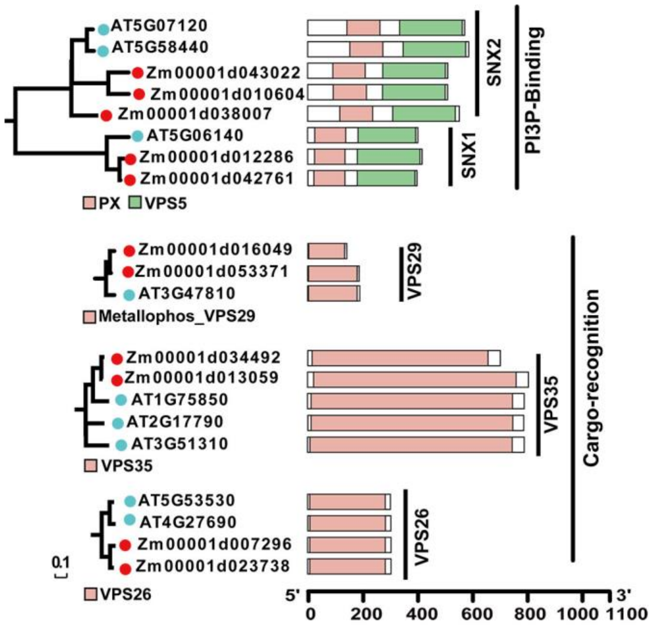

Supplementary Figure S7. Phylogenetic and domain analysis of various components of Retromer complex from *Arabidopsis* and maize. The full-length amino acid sequences of factors were aligned by ClustalW, and phylogenetic trees were created using MEGA (version 6.0) with the Neighbor-Joining method (bootstrap value set at 1,000). Protein domains were visualized by TBtools software (v1.09854). Evolview-v2 software was used for visualization phylogenetic trees. The cyan and red dots indicate protein from *Arabidopsis* and maize, respectively. At, *Arabidopsis thaliana*; Zm, *Zea mays*. Tree scale: 0.1.

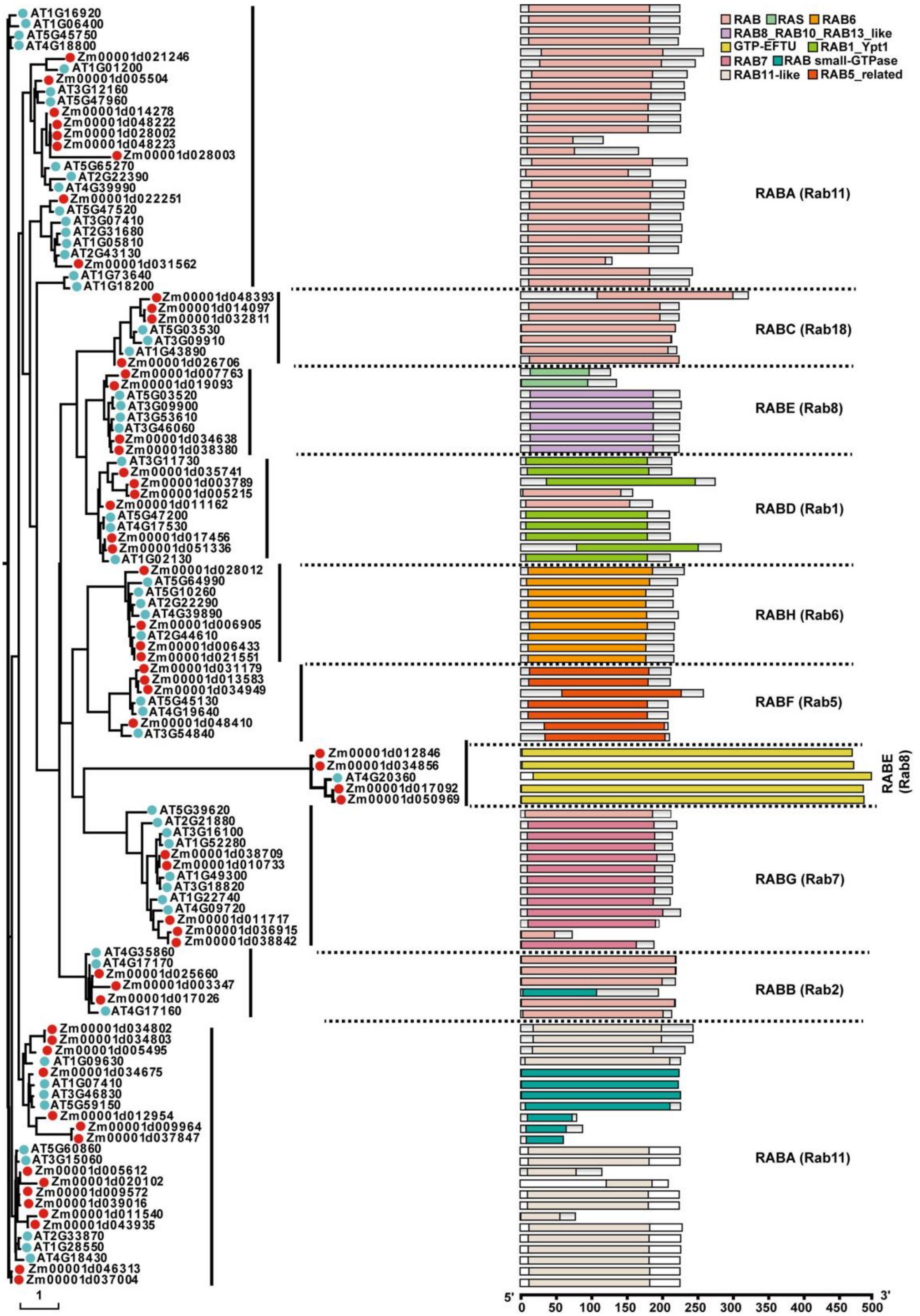

Supplementary Figure S8. Phylogenetic and domain analysis of various Rab proteins from *Arabidopsis* and maize. The full-length amino acid sequences of factors were aligned by ClustalW, and phylogenetic trees were created using MEGA (version 6.0) with the Neighbor-Joining method (bootstrap value set at 1,000). Protein domains were visualized by TBtools software (v1.09854). Evolview-v2 software was used for

visualization phylogenetic trees. The cyan and red dots indicate protein from *Arabidopsis* and maize, respectively. At, *Arabidopsis thaliana*; Zm, *Zea mays*. Tree scale: 1.

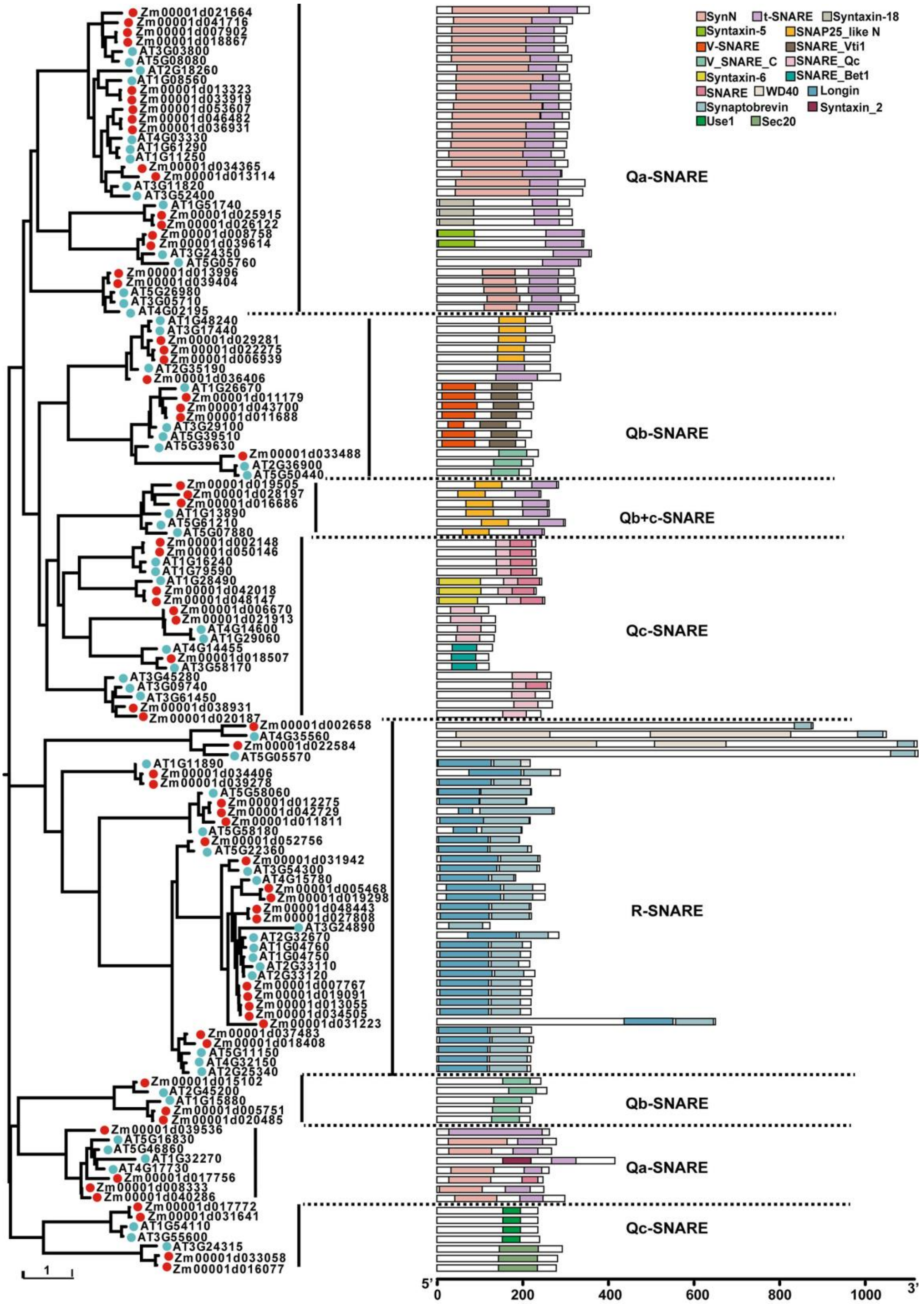

Supplementary Figure S9. Phylogenetic and domain analysis of various SNARE proteins from *Arabidopsis* and maize. The full-length amino acid sequences of factors were aligned by ClustalW, and phylogenetic trees were created using MEGA (version 6.0) with the Neighbor-Joining method (bootstrap value set at 1,000). Protein domains were visualized by TBtools software (v1.09854). Evolview-v2 software was used for

visualization phylogenetic trees. The cyan and red dots indicate protein from *Arabidopsis* and maize, respectively. At, *Arabidopsis thaliana*; Zm, *Zea mays*. Tree scale: 1.

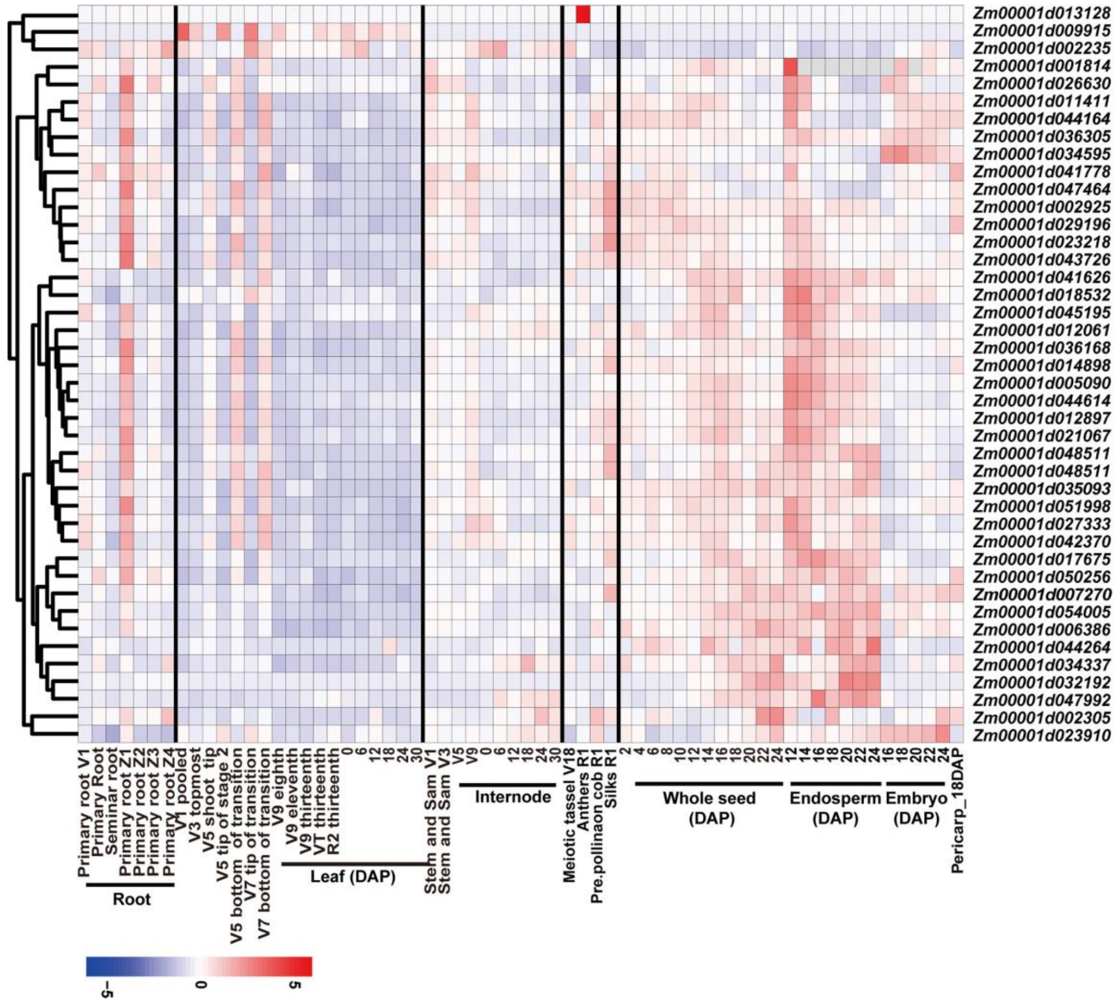

Supplementary Figure S10. The spatio-temporal expression profiles of maize genes encoding components of Clathrin-Coated protein complex and adaptor complexes. The expression of genes involved in Clathrin-Coated protein complexes and Adaptor complexes is analyzed based on the reads per kilobase per one million reads (RPKM) of 64 maize tissue specific samples. The blue, white, and red colors indicate the low, medium, and high gene expression values, respectively.

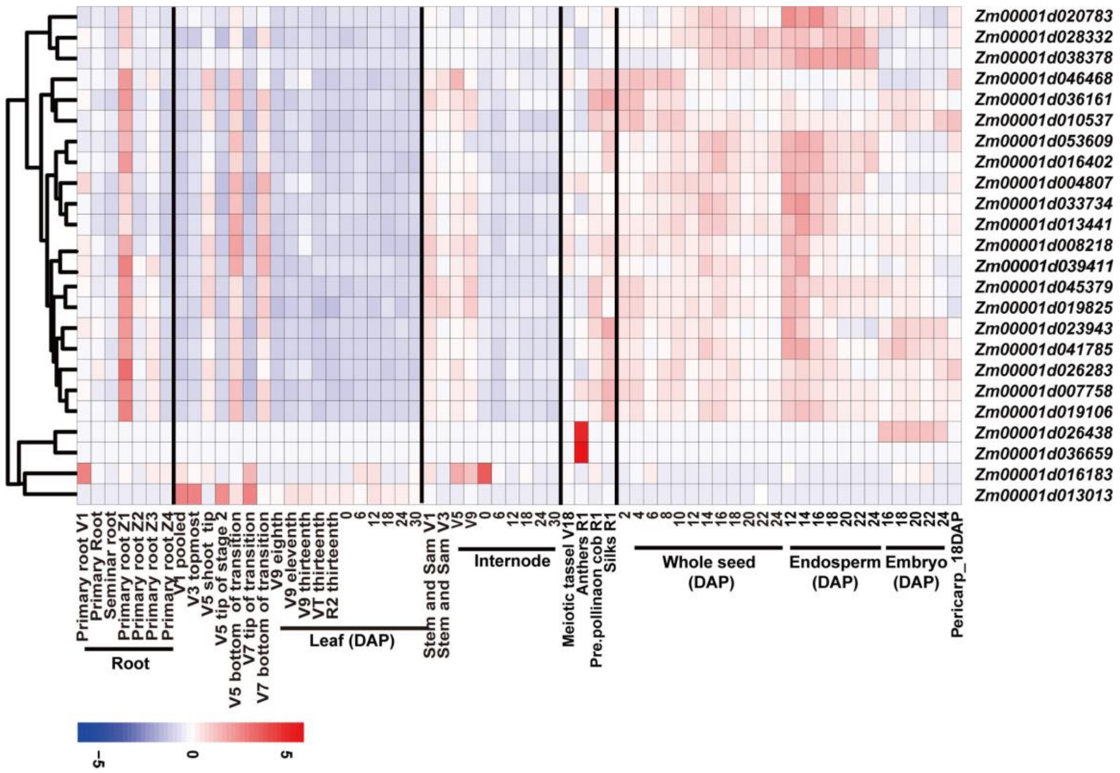

Supplementary Figure S11. The spatio-temporal expression profiles of maize genes encoding components of COP-I complex. The expression of genes involved in COP-I complex is analyzed based on the reads per kilobase per one million reads (RPKM) of 64 maize tissue specific samples. The blue, white, and red colors indicate the low, medium, and high gene expression values, respectively.

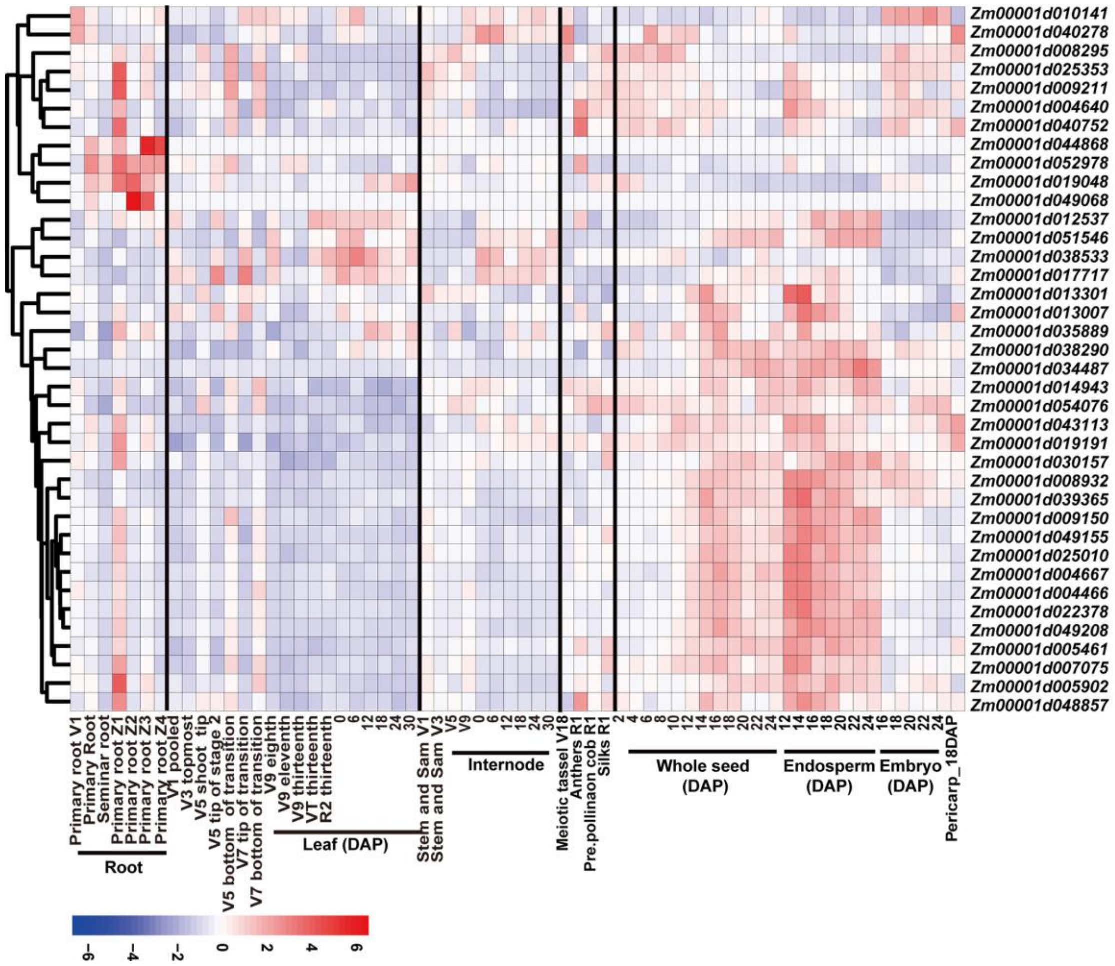

Supplementary Figure S12. The spatio-temporal expression profiles of maize genes encoding components of COP-II complex. The expression of genes involved in COP-II complex is analyzed based on the reads per kilobase per one million reads (RPKM) of 64 maize tissue specific samples. The blue, white, and red colors indicate the low, medium, and high gene expression values, respectively.

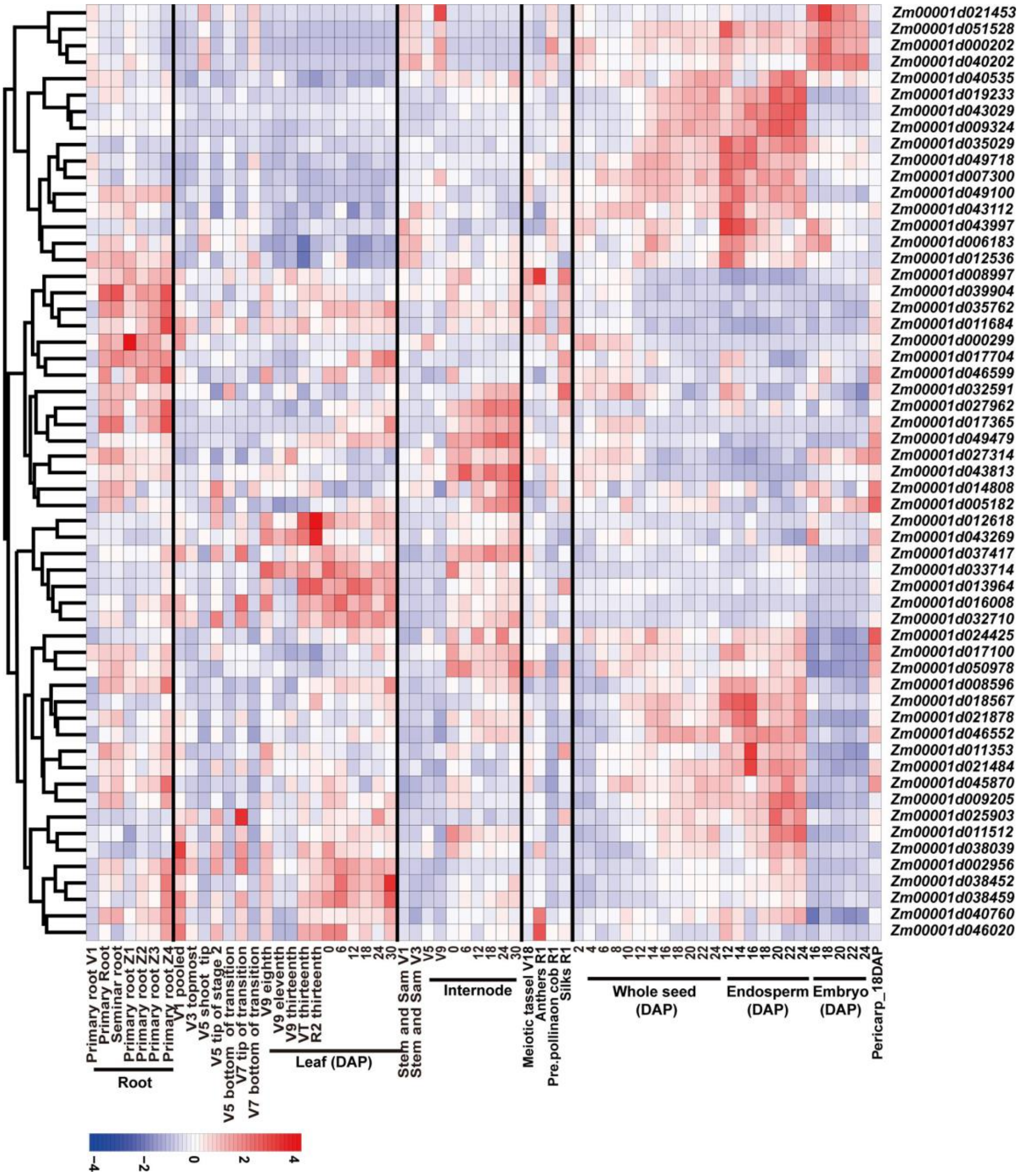

Supplementary Figure S13. The spatio-temporal expression profiles of maize genes encoding components of ESCRT machinery. The expression of genes involved in ESCRT machinery is analyzed based on the reads per kilobase per one million reads (RPKM) of 64 maize tissue specific samples. The blue, white, and red colors indicate the low, medium, and high gene expression values, respectively.

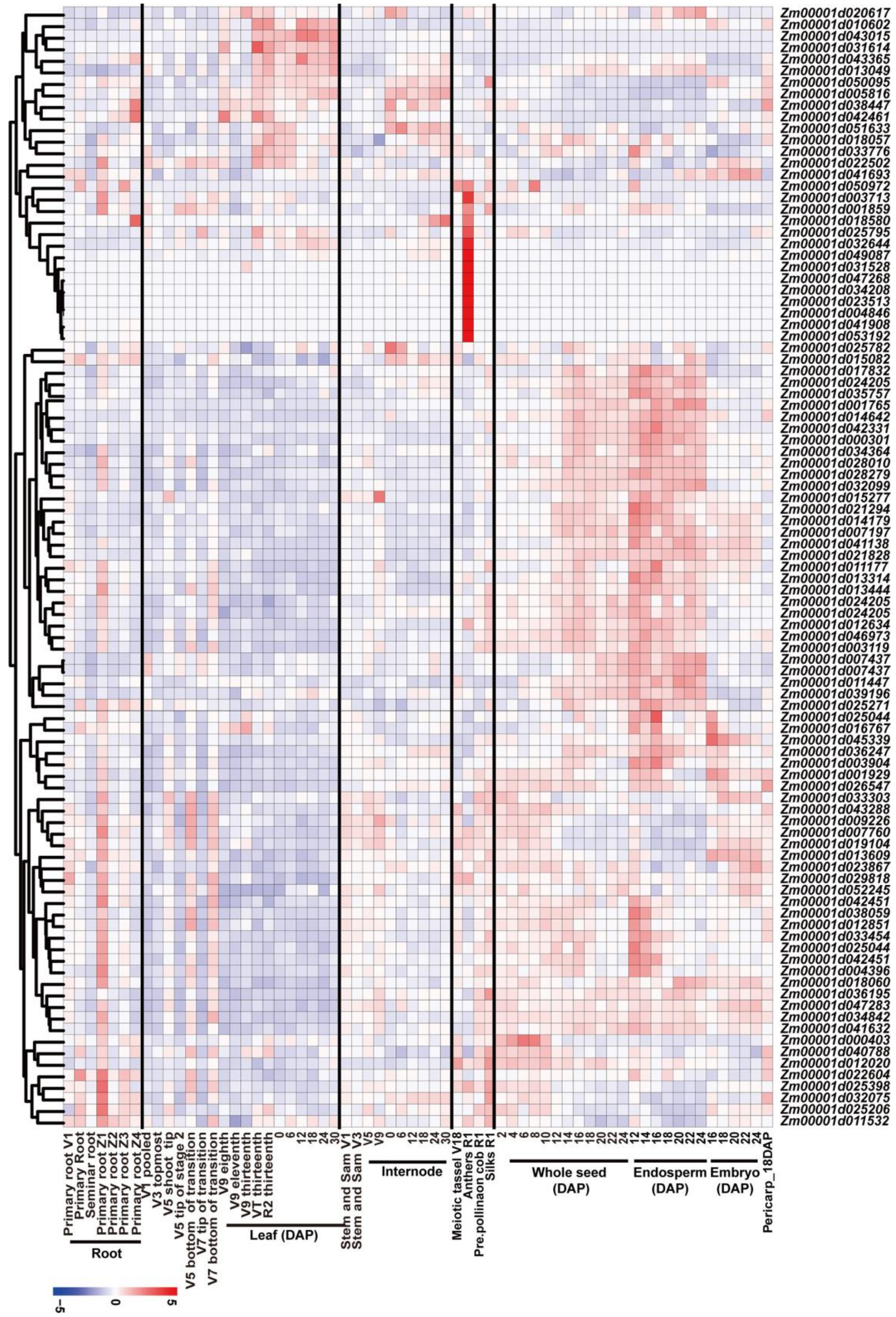

Supplementary Figure S14. The spatio-temporal expression profiles of maize genes encoding components of Tethering complexes. The expression of genes involved in Tethering complexes is analyzed based on the reads per kilobase per one million reads (RPKM) of 64 maize tissue specific samples. The blue, white, and red colors indicate

the low, medium, and high gene expression values, respectively.

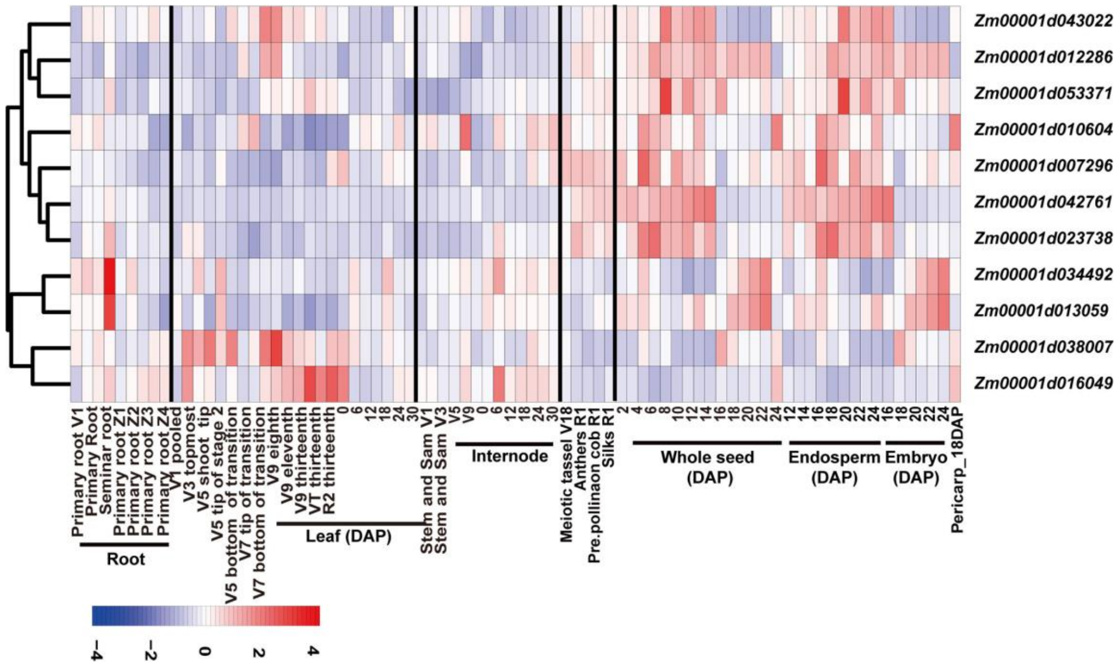

Supplementary Figure S15. The spatio-temporal expression profiles of maize genes encoding components of Retromer complex. The expression of genes involved Retromer complex is analyzed based on the reads per kilobase per one million reads (RPKM) of 64 maize tissue specific samples. The blue, white, and red colors indicate the low, medium, and high gene expression values, respectively.

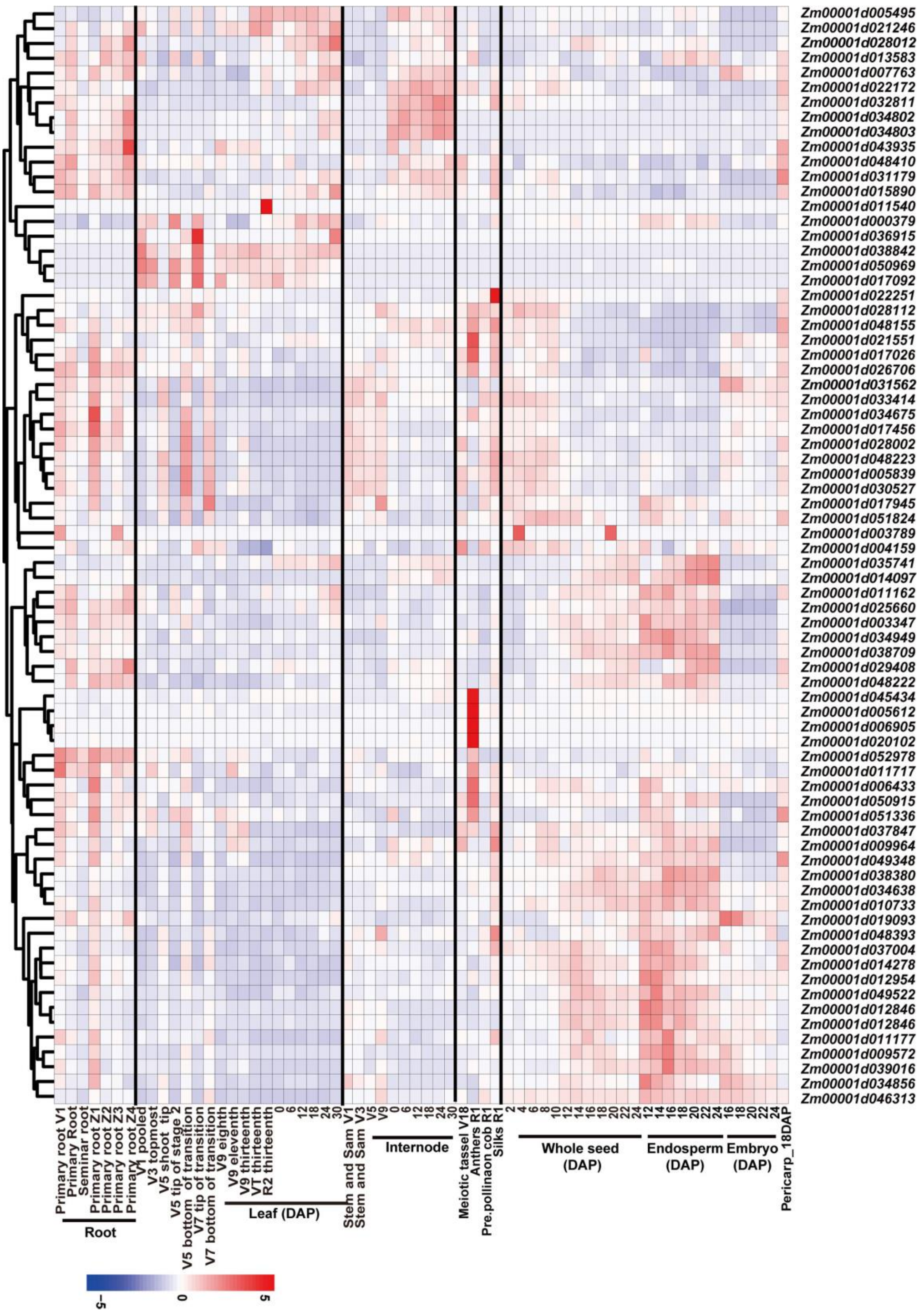

Supplementary Figure S16. The spatio-temporal expression profiles of maize genes encoding various Rab proteins. The expression of genes encoding Rab GTPases is analyzed based on the reads per kilobase per one million reads (RPKM) of 64 maize tissue specific samples. The blue, white, and red colors indicate the low, medium, and high gene expression values, respectively.

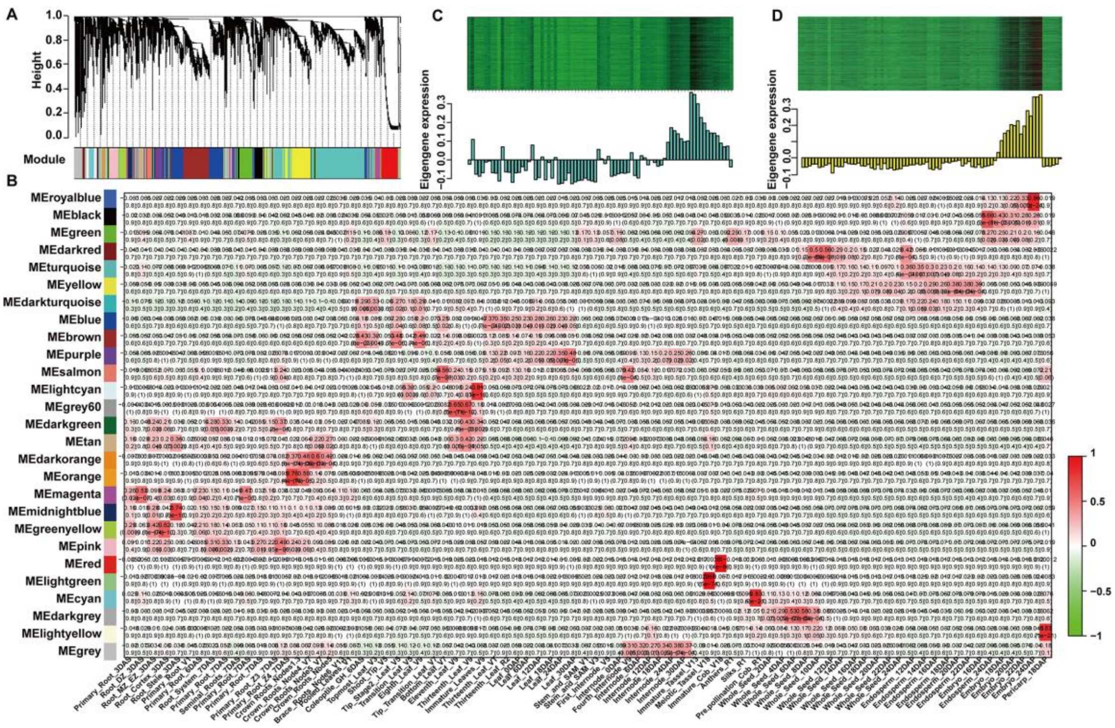

Supplementary Figure S17. Weighted gene co-expression network analysis (WGCNA) of the spatial and temporal patterns of maize genes. **(A)** The WGCNA is conducted with a total of 3,0705 genes expressed in 80 different tissues, and co-expressed genes were clustered into modules indicated by different color bars beneath each dendrogram. **(B)** Module-trait correlations and corresponding *p*-values. The color scale indicates the module-trait correlation from -1 (green) to 1 (red). **(C)** Heatmap and bar plot of module turquoise. The heatmap (upper panel) indicates the expression level of genes in the module, and the bar plot (lower panel) indicates the module eigengene relative expression level. **(D)** Heatmap and bar plot of module yellow. The heatmap (upper panel) indicates the expression level of genes in the module and the bar plot (lower panel) indicates the module eigengene relative expression level.

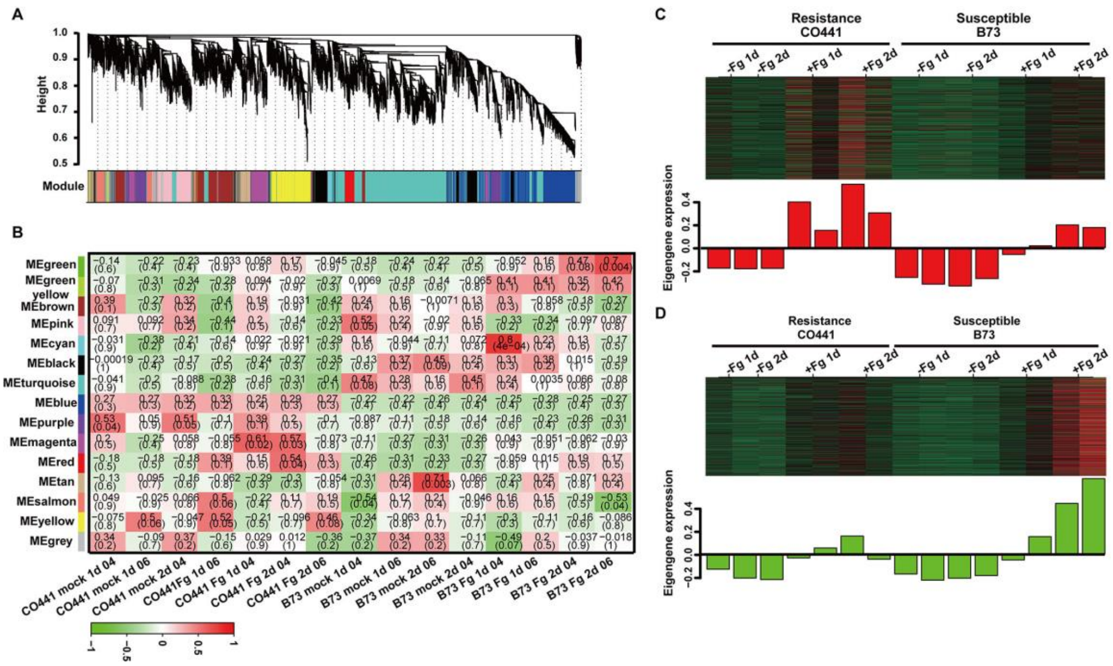

Supplementary Figure S18. Weighted gene co-expression network analysis (WGCNA) of the expression patterns of maize gene responding to Gibberella ear rot disease. **(A)** The WGCNA is conducted on a total of 12,528 genes expressed in resistant line CO441 and susceptible line B73 during pathogen infection. The co-expressed genes are clustered into modules indicated by different color bars beneath each dendrogram. **(B)** Module-trait correlations and the corresponding p-values. The color scale indicates the module-trait correlation from -1 (green) to 1 (red). **(C, D)** Heatmaps and bar plots of module red and green. The heatmaps indicate the gene expression levels, and the bar plots indicate the module eigengene relative expression levels.

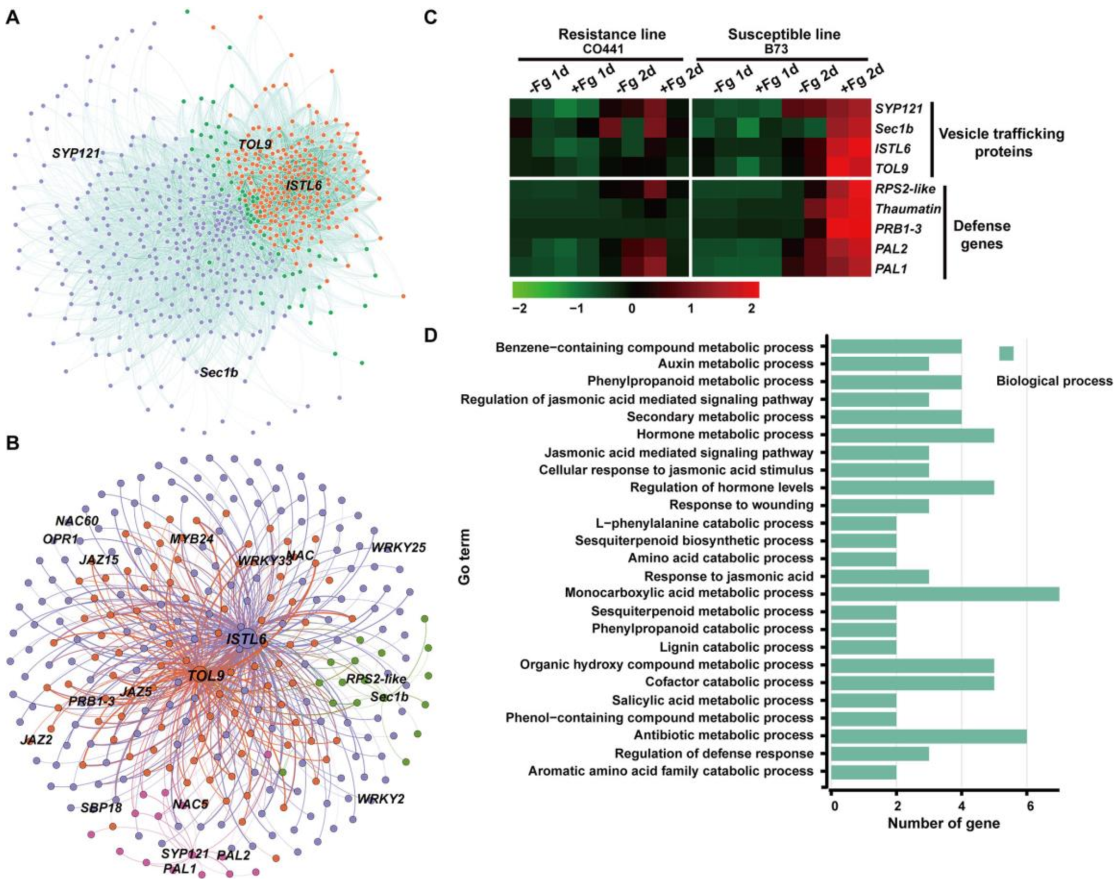

Supplementary Figure S19. Co-expression network of membrane trafficking genes responding to Gibberella ear rot disease in two maize inbred lines. **(A)** Co-expression network of module green. Nodes are color coded based on consensus modules identified by R language WGCNA package. Edges are constructed between genes. **(B)** The sub-network visualization for membrane trafficking genes in module green with correlation coefficient (*r*) > 0.3. **(C)** Expression profiles of endomembrane system genes and defense genes identified in module green during Gibberella infection. The values in red and green indicate gene increases and decreases in expression, respectively. **(D)** Gene Ontology (GO) enrichment analysis of the sub-network co-expressed genes.

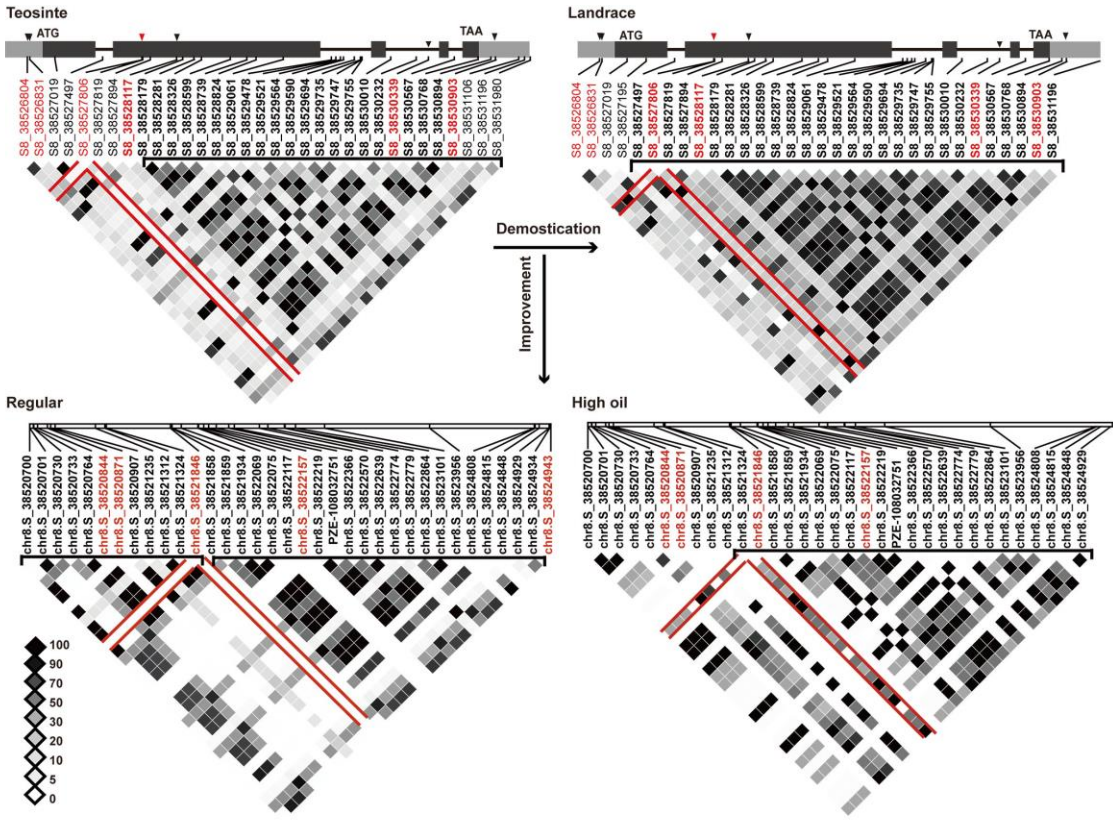

Supplementary Figure S20. Gene structure and linkage disequilibrium (LD) patterns of *ZmSec23a* locus. SNPs (MAF ≥ 0.05) of candidate genes are used to calculate the square values of correlation coefficient (r^2) to measure LD using HaploView v4.2. The gene diagram, dark and grey boxes represent exons and UTRs, respectively.

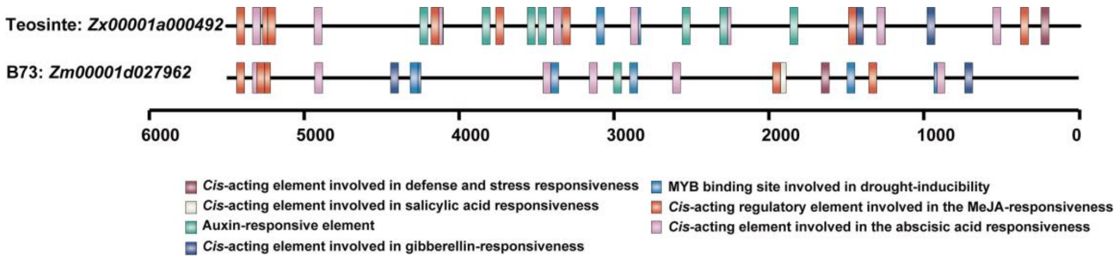

Supplementary Figure S21. Distribution of stress-related *cis*-regulatory elements in the promoter regions of *ZmVPS37A* of B73 inbred line and teosinte. All cis-regulatory elements are identified by PLACE (https://www.dna.affrc.go.jp/PLACE/?action=newplace) and indicated by different color bar. The annotation of each element is listed beneath the promoters. TBtools software was used for visualization.
